# Biological Significance of Photoreceptor Photocycle Length: VIVID Photocycle Governs the Dynamic VIVID-White Collar Complex Pool Mediating Photo-adaptation and Response to Changes in Light Intensity

**DOI:** 10.1371/journal.pgen.1005215

**Published:** 2015-05-15

**Authors:** Arko Dasgupta, Chen-Hui Chen, ChangHwan Lee, Amy S. Gladfelter, Jay C. Dunlap, Jennifer J. Loros

**Affiliations:** 1 Department of Genetics, Geisel School of Medicine, Dartmouth, Hanover, New Hampshire, United States of America; 2 Department of Biological Sciences, Dartmouth, Hanover, New Hampshire, United States of America; 3 Department of Biochemistry, Geisel School of Medicine, Dartmouth, Hanover, New Hampshire, United States of America; Cornell University, UNITED STATES

## Abstract

Most organisms on earth sense light through the use of chromophore-bearing photoreceptive proteins with distinct and characteristic photocycle lengths, yet the biological significance of this adduct decay length is neither understood nor has been tested. In the filamentous fungus *Neurospora crassa* VIVID (VVD) is a critical player in the process of photoadaptation, the attenuation of light-induced responses and the ability to maintain photosensitivity in response to changing light intensities. Detailed *in vitro* analysis of the photochemistry of the blue light sensing, FAD binding, LOV domain of VVD has revealed residues around the site of photo-adduct formation that influence the stability of the adduct state (light state), that is, altering the photocycle length. We have examined the biological significance of VVD photocycle length to photoadaptation and report that a double substitution mutant (*vvdI74VI85V*), previously shown to have a very fast light to dark state reversion *in vitro*, shows significantly reduced interaction with the White Collar Complex (WCC) resulting in a substantial photoadaptation defect. This reduced interaction impacts photoreceptor transcription factor WHITE COLLAR-1 (WC-1) protein stability when *N*. *crassa* is exposed to light: The fast-reverting mutant VVD is unable to form a dynamic VVD-WCC pool of the size required for photoadaptation as assayed both by attenuation of gene expression and the ability to respond to increasing light intensity. Additionally, transcription of the clock gene *frequency* (*frq*) is sensitive to changing light intensity in a wild-type strain but not in the fast photo-reversion mutant indicating that the establishment of this dynamic VVD-WCC pool is essential in general photobiology and circadian biology. Thus, VVD photocycle length appears sculpted to establish a VVD-WCC reservoir of sufficient size to sustain photoadaptation while maintaining sensitivity to changing light intensity. The great diversity in photocycle kinetics among photoreceptors may be viewed as reflecting adaptive responses to specific and salient tasks required by organisms to respond to different photic environments.

## Author Summary

Sensing light from the environment using a variety of photoreceptors is of great adaptive significance for most eukaryotes. A key feature of photoreceptors is the photocycle length, the time taken to decay from the initial signaling light state back to the receptive dark state; however, the significance of photocycle length, or adduct decay length, has not been tested in a biological setting. The photocycle length is determined by the chemical environment of the active site where a photon absorbing chromophore forms an adduct with a conserved amino acid. There is clear evidence of evolutionary selection for a particular photocycle length even between photoreceptors containing the same prototypic light-sensing domains suggesting functional relevance. Using defined *in vitro* mutations that change the photocycle length of the VIVID (VVD) protein over 4 orders of magnitude we were able to ascribe a pivotal role of the native photochemistry of the protein in its function as a photoreceptor in the light and circadian biology of *Neurospora crassa*. This study links *in vitro* photochemical studies with *in vivo* function and provides evidence that the true evolutionary and functional significance of native photochemistry of photoreceptors can be enhanced by studying photocycle mutants in their native systems.

## Introduction

Most organisms and nearly all eukaryotes respond to light in their environment, and do so through the use of proteins specially adapted to respond to light. Such photoreceptor proteins most often sense light through the use of prosthetic groups, chromophores, chosen by evolution for their ability to absorb light of particularly relevant wavelengths, flavins for UV-A and blue light, trans-p-coumaric acid for yellow, retinals for green, and tetrapyrroles for red and infrared [1]. Absorption of light elicits photochemical changes in a chromophore resulting in conformational changes in the photoreceptor protein that initiate the intracellular signaling leading to a biological response, while at the same time leaving the photoreceptor itself unable to respond to a second light stimulus. In most cases, however, this loss-of-response is reversible through photochemistry [2,3] or via a photocycle in which thermal decay of the activated state restores the receptor to the ground (receptive) state. The kinetics of a particular photocycle is highly variable both among classes of photoreceptor domains and even within a class of photoreceptor domains. Although the general biochemistry of photoreception is well understood [1] and insights into the determinants of photocycle length are emerging as described below, much less is known regarding the functional and adaptive significance of the wide range of known photocycle lengths.

The structural basis of adduct decay length has been probed in great detail among photoreceptor proteins using LOV (Light, Oxygen, Voltage)-domains to sense blue light as commonly found in bacteria, plants and fungi [4–10]. These domains bind a flavin (FMN or FAD) that when photoactivated associates with, usually covalently, the LOV domain causing it to undergo a light induced conformational change [11–13]; propagation of the structural change within the photoreceptor initiates signaling that leads to the photoresponse [11,14–17]. Intriguingly, although the initial photochemical reaction (light-induced adduct formation at conserved residues within a LOV domain) is virtually identical in all LOV domains examined, the lifetime of the photo-adduct signaling state shows extremely broad variation. For example, the photo-adduct stability of fungal (e.g. WC-1 and VVD) and bacterial (e.g. YtvA) LOV domains ranges from hours to days [4,16,18–20] whereas the photo-adduct stability of plant Phototropin-1 LOV2 domains is on the order of seconds [21,22]. Recent studies have begun to elucidate the structural bases of photocycle length among LOV domain proteins [3,23,24], including studies focused on VVD [20] which governs photoadaptation in *Neurospora* [19,25], providing the ideal model for examining the adaptive significance of photocycle length.

Vivid (VVD) is a blue light photoreceptor protein consisting of a LOV domain and an N-terminal cap [16,26] and displays prototypic reversible changes upon light activation [27]. Expression of VVD itself is light-induced [26] through the action of the photoreceptive transcription factor WC-1 in association with WC-2, the White Collar Complex (WCC) [28–30]. Light activates WC-1 and the WCC drives the expression of hundreds of light-responsive genes when *N*. *crassa* is exposed to blue light [31–33]; light-activation also destabilizes WC-1 which is lost via phosphorylation-associated turnover [28]. Light-activated VVD dimerizes *in vitro* and also interacts with PAS domains in the WCC *in vivo* [25,27,34–36]. Light-activation and subsequent conformational changes have been shown to be important for VVD’s primary function which is to interact with and attenuate the transcriptional activity of the WCC [16,26,37]. This explains the biological role of VVD in the cell, to modulate phase-setting of the Neurospora circadian clock that is initiated by the WCC [26,38] and to attenuate light-induced gene expression and regulate responses to changing intensities of light (photoadaptation) [19,25]. Recent structural and biochemical analyses of the VVD protein have provided molecular details of the determinants impacting photocycle kinetics of this protein [16,20,27], revealing that the *in vitro* adduct decay length of VVD is remarkably plastic and can be adjusted over four orders of magnitude – from 28 seconds to 50 hours- primarily by influencing the chemical environment of the active photo-adduct formation site [20]. Given that the light-response and the core-circadian machinery in *N*. *crassa* are well defined [39,40] and VVD’s role in these processes is appreciated, we applied knowledge from the recent structural advances in a biological context to see how an altered photocycle length as defined *in vitro* might influence biological responses *in vivo*. The surprising results show that VVD photocycle length plays a dominant role in determining the utility of this photoreceptor such that mutants with inappropriately fast photocycles display severe defects in photoadaptation, this despite their ability to sense and respond to light, and also display impaired circadian rhythmicity under quasi-normal light-dark cycles. In a broader sense the results suggest strong selective pressure to adjust photocycle length to specific biological tasks, and in turn imply that photocycle length can be informative regarding the mechanism of the intracellular task for which a photoreceptor has evolved.

## Results

### A photoadaptation phenotype is associated with the alteration of the VVD photocycle

**Fig 1:**
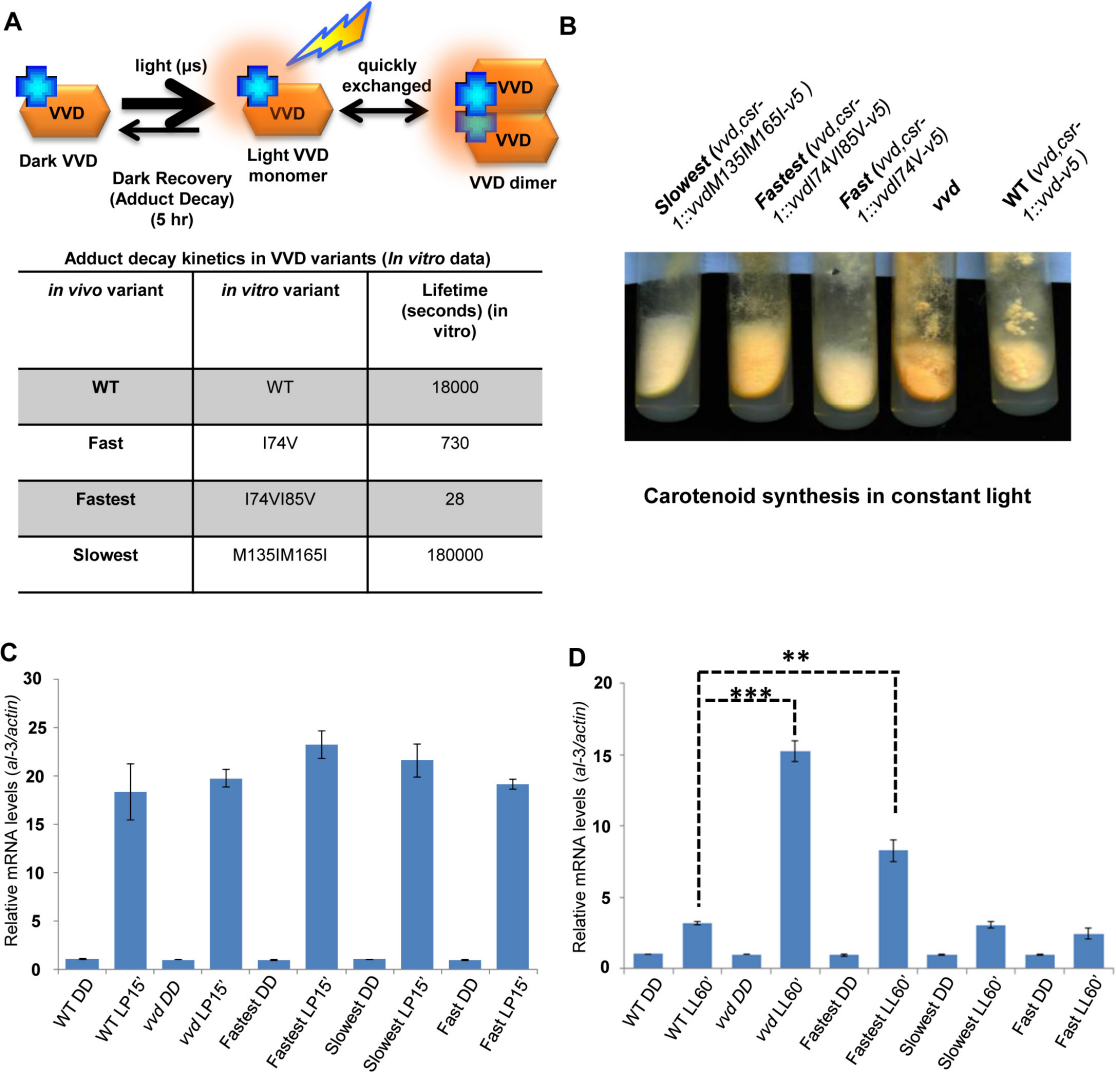
Altering VIVID (VVD) photocycle through site-directed mutagenesis reveals a photoadaptation mutant. (A) Cartoon showing *in vitro* photochemistry of VVD light-activated dimer and the influence of specific mutations on the photo-adduct stability/life-time [20]. Blue crosses represent chromophores, and light activated chromophores are shown with orange halo. (B) WT, *vvd* null and three photocycle length variants were grown on solid minimal medium slants, exposed to constant light (40 μM m^-2^s^-1^) for 4–5 days and the mutants visually compared to the WT for carotenoid biosynthesis. (C) Strains (n = 3) exposed to a 15 minute light pulse (LP15’) were subjected to RT-PCR to determine *al*-3 gene expression levels as a measure of the integrity of the light response in the photocycle mutants. (D) Strains (n = 3) were exposed to bright white light for 60 minutes (LL60’) to study photoadaptation response using *al*-3 gene expression as a readout. The fastest photocycle mutant shows a partial loss of photoadaptation at the gene expression level when compared to the WT strain as seen by the higher levels of *al*-3 mRNA after 60 minutes of light exposure (LL60’). Asterisks indicate statistical significance as determined by an unpaired *t* test. **P<0.01, ***P<0.001.

The normal photocycle length of VVD is 5 hours, and Zoltowski *et al*.[20] have described adduct decay rates of twenty distinct mutants that cause VVD to cycle faster or slower *in vitro*. Choosing three of the most extreme, we carried out *in vivo* functional, genetic and biochemical analysis of *vvdI74V* and *vvdI74VI85V* whose photocycles are reduced to 730 seconds and 28 seconds respectively, and *vvdM135IM165I* whose photocycle is lengthened to 50 hours [20]. Deprotonation (via a base in solvent, or the conserved glutamine, or Cys 108) of N_5_ in the flavin isoalloxazine ring, in adduct state with Cys108, regulates the lifetime of the adduct state. The I->V substitutions at position 74 and 85 leads to the steric destabilization of the photo-adduct state by increasing the solvent accessibility of the active site (Cys 108). The M->I substitutions at 135 and 165 position alter the steric and electronic environment (electron rich methionine changed to relatively electron poor aliphatic amino acids) of the flavin molecule to lead to stabilization of the photo-adduct state. To test whether mutations altering the photochemical properties of the VVD photocycle (**Fig 1A**) influence VVD function *in vivo*, DNA constructs encoding the V5 tagged (C-terminal) versions of the wild-type (WT) and mutant VVD protein were targeted to the *cyclosporin-resistance-1* (*csr-1)* locus in a *vvd* gene deletion [25,41] strain (*vvd* null strain). For simplicity we will henceforth call the *vvd* null,*csr-1*::*vvdI74V-v5* strain as the fast photocycle mutant, the *vvd* null,*csr-1*::*vvdI74VI85V-v5* strain as the fastest photocycle mutant, the *vvd* null,*csr-1*::*vvdM135IM165I-v5* strain as the slowest photocycle mutant and the *vvd* null,*csr-1*::*vvd-v5* strain as the WT strain (**Fig 1A**). The fastest photocycle mutant displays a hyper-carotenoid synthesis phenotype when exposed to constant bright light for 4–6 days (**Fig 1B**). This phenotype is similar to but not as intense as the *vvd* null strain which is characterized by bright orange coloration of its hyphae when exposed to constant bright light, indicating a partial loss of photoadaptation in the fastest mutant. No significant color phenotype was observed with the fast and the slowest photocycle mutants. To test if the phenotype is a result of an aberrant light response we exposed our mutants and the WT strain to a 15 minute white light (~40 μM m^-2^ s^-1^) pulse (LP15’) and studied the mRNA levels of the *al*-3 *(albino*-3) gene. The *al*-3 gene encodes geranylgeranyl pyrophosphate synthase in the carotenoid biosynthetic pathway and is expressed immediately after *N*. *crassa* is exposed to light [42]. We found the *al*-3 gene expressed to similar levels in all mutants as well as the WT strain after a light pulse (**Fig 1C**) suggesting that the initial light response driven by the WCC is intact in all these strains. We then exposed the strains to 60 minutes of white light (LL60’) and studied *al*-3 mRNA levels in order to test if the phenotype of the fastest photocycle mutant was indeed due to a partial loss of photoadaptation. In *N*. *crassa* the levels of *al*-3 mRNA drop significantly after 60 minutes of constant light exposure as a result of a photoadaptation mechanism where VVD interacts with WCC and inhibits its transcriptional activity [25,35,36]. As anticipated from the observed carotenogenesis phenotype, the fastest photocycle mutant shows a partial loss of photoadaptation in this assay. As expected *al*-3 mRNA levels show no repression in the *vvd* null control while the fastest photocycle strain displays a partial repression compared to the WT strain after 60 minutes of exposure (LL60’) to white light (**Fig 1D**). We examined VVD protein synthesized after 60 minutes of light exposure by Western analysis, finding all strains expressing equivalent amounts of VVD protein (**S1A Fig**), demonstrating that the observed phenotype was not due to differences in the amounts of VVD. Additionally, we tested other light-induced genes including *sub*-1, *cryptochrome* and *al*-1 and observed the same partial loss of photoadaptation in the fastest photocycle mutant as seen in the case of the *al*-3 gene (**S1B, S1C and S1D Fig**).

### The photoadaptation defect is independent of light intensity and photoreversion and is not a result of aberrant sub-cellular localization

To further characterize the phenotype we examined photoadaptation under a very low light intensity (~1.5 μM m^-2^s^-1^) of blue light as well as monitored the cellular localization of the VVD mutants. Blue light was used here to remove the effect of light-driven photoreversion of activated VVD [2,3] that has been a confounding variable in all prior work on VVD. Briefly, the photoactivated form of the LOV domain photoreceptors retains the covalently bound FAD. When this chromophore absorbs near-UV light, which constitutes a portion of the white light used in all prior studies, some of the light-activated VVD is reverted to the dark state [2,3]. As a result, under white light VVD is a mixture of dark state and light (activated) state whose proportions reflect both light intensity and the inherent thermal stability of the activated form. Blue wavelengths of light do not result in light driven reversion [2] so the effect of photocycle length can be studied without this uncontrolled variable.

**Fig 2:**
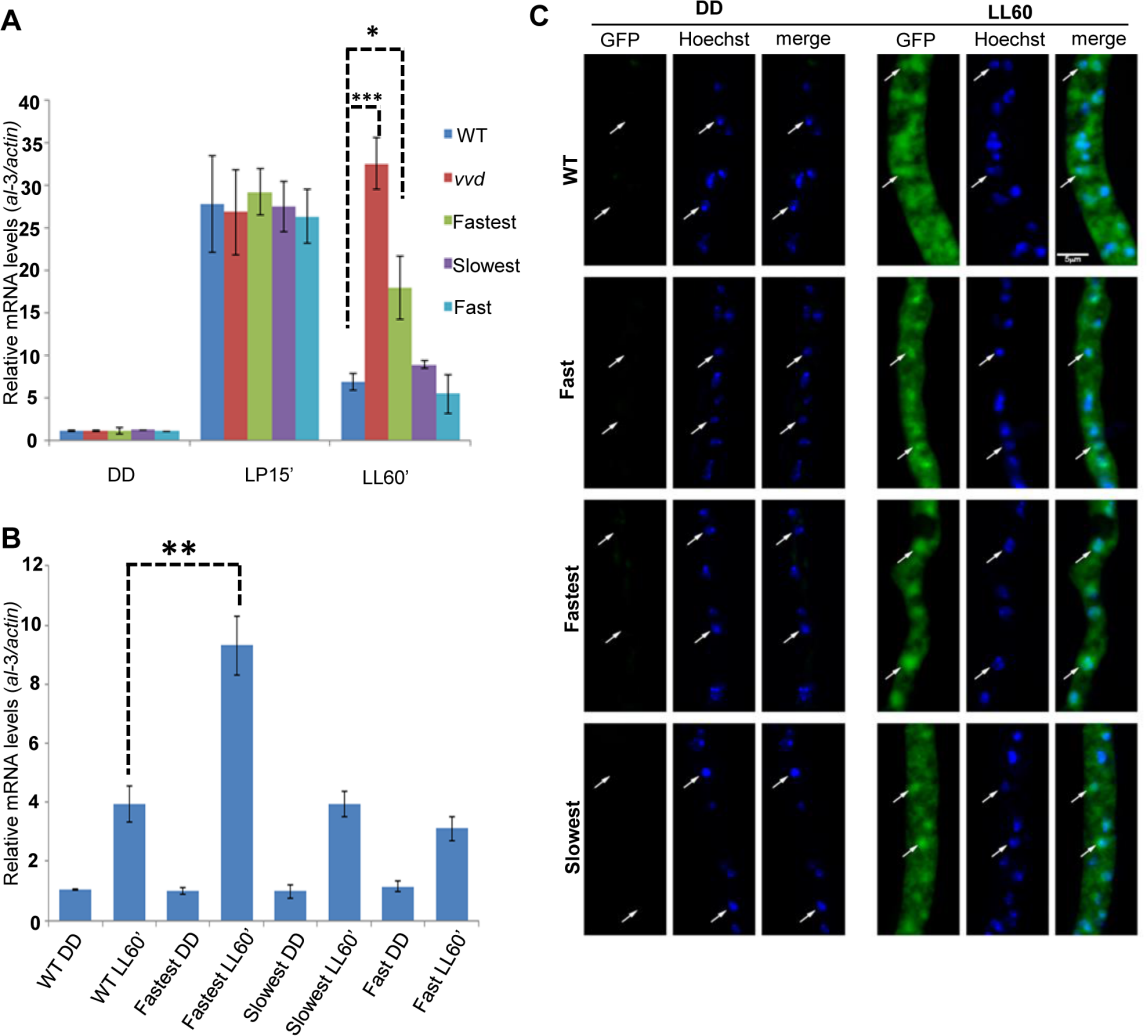
Photoadaptation defect exists at low light intensity and mutations do not effect nuclear localization of VVD. (A) Strains (n = 3) were exposed (after 48 hours in dark) to very low light intensity blue light (~1.5 μM m^-2^s^-1^ of blue light) for 15 minutes and 60 minutes to study light response and photoadaptation via *al*-3 gene expression. (B) N-terminal GFP tagged VVD mutant strains were created for sub-localization studies and photoadaptation defect was tested after 60 minute bright white light(40 μM m^-2^s^-1^) exposure in these strains using *al*-3 expression as readout (n = 3). (C) Fixed cell microscopy qualitatively shows that the mutants retain the ability to localize VVD protein to the nucleus after exposure to white light (40 μM m^-2^s^-1^) induces expression. Asterisks indicate statistical significance as determined by an unpaired *t* test. *P<0.05, **P<0.01, ***P < 0.001.

Under low intensity blue light the fastest photocycle mutant again showed a partial loss of photoadaptation after a 60 minute exposure (**Fig 2A**) suggesting that the defect does not require chromophore saturation. It has been previously shown that VVD localizes to the nucleus after synthesis independent of light-exposure [25] and hence is in a subcellular compartment where it can interact with the WCC and bring about photoadaptation [25,35,36]. To confirm the VVD mutations were not interfering with nuclear localization of VVD, we engineered an additional GFP tag (N-terminal) to the constructs and conducted microscopic subcellular localization studies as previously described [25]. The GFP-tagged fastest photocycle mutant strain recapitulated the phenotype (**S2 Fig**) shown before with the V5-tagged version, showing a partial loss of photoadaptation at the level of gene expression (**Fig 2B**). We found that the GFP-tagged VVD mutants were able to localize to the nucleus at levels comparable to the GFP-tagged WT protein (**Fig 2C**).

### The fastest photocycle mutant is unable to inhibit WCC activity due to reduced VVD-WCC interaction leading to the partial loss of photoadaptation

**Fig 3:**
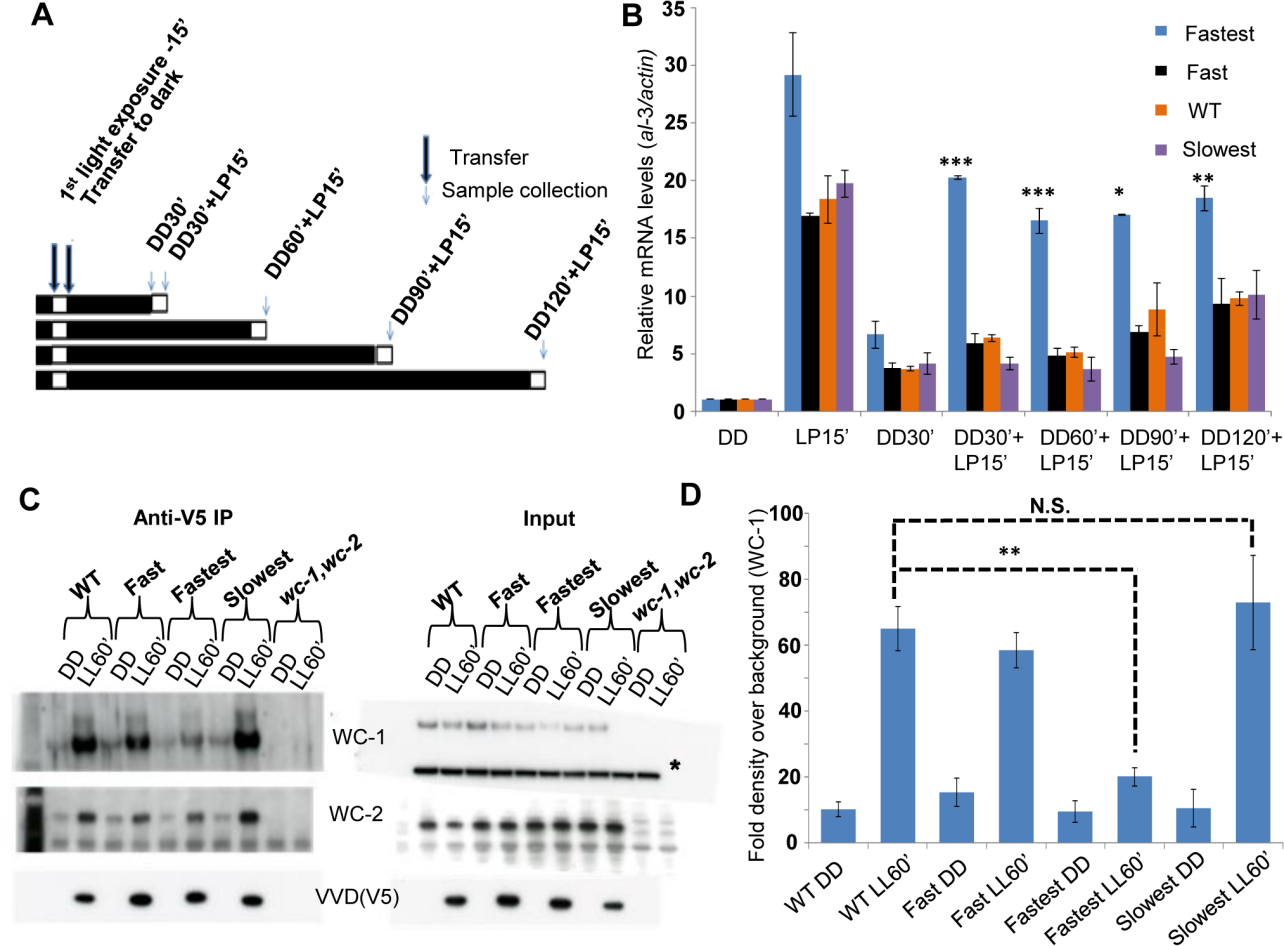
The fastest photocycle mutant is unable to inhibit White Collar Complex (WCC) activity due to reduced VVD-WCC interaction. (A) Two-pulse experimental design for testing WCC transcriptional activity in response to a second light pulse. Strains (n = 3) were exposed to a 15 minute white light pulse (LP15’, 40 μM m-2s-1) resulting in the induction of VVD then followed by dark transfer (DD) and a subsequent 15 minute light pulse (LP15’, 40 μM m-2s-1) after various times in the dark (DD30’,DD60’, DD90’, DD120’). (B) WCC activity after second light pulse as determined by *al*-3 gene expression shows that the fastest photocycle mutant is able to reactivate WCC to a greater extent when compared to WT and other mutants. (C) VVD-WCC interaction using DSP cross-linking under white light (40 μM m-2s-1) conditions [25]. VVD-V5 co-IP pull down products (WCC and VVD) were analyzed using Western Blotting techniques. Membranes for Anti-V5 IP were developed using more sensitive Femto reagent and the *wc-1*,*wc-2* null strain was used as a background control for the Western Blots. (D) Blots (n = 3) were quantified by densitometry using NIH Image J software to measure VVD-WCC interaction in the WT and mutants. * non-specific band/ loading control on Western blots. Asterisks indicate statistical significance when compared with WT as determined by an unpaired *t* test. ***P < 0.001, **P<0.01 *P<0.05, N.S.- difference not significant.

The primary function of the VVD protein is to physically interact with WCC and bring about repression of transcriptional activity resulting in photoadaptation [25,35,36]. We tested if VVD-WCC interaction was altered in the mutants when compared to the WT strain. We first exposed the strains to a 15 minute white light pulse (LP15’) to generate VVD in the system. This was followed by transfer to dark for various times (allowing dark-reversion) before exposing the cultures to a second 15 minute light pulse (**Fig 3A**), a so-called 2-pulse experiment. The rationale was if the mutants had increased or decreased interaction between VVD and WCC a difference in the response (WCC transcriptional activity) to the second light pulse would be expected. The fastest photocycle mutant shows significantly higher WCC activity on exposure to the second light pulse after dark exposures from 30 to 120 minutes, as assayed by *al*-3 mRNA levels compared to other mutants and the WT strain (**Fig 3B**). This suggested that VVD-WCC interaction is impaired in the fastest photocycle mutant and that VVD protein in this mutant is unable to inhibit WCC transcriptional activity in response to the second light pulse. To directly test the physical interaction between WCC and VVD in the mutants we performed a previously described DSP cross-linking co-immunoprecipitation assay [25] to study the amount of WCC that was bound to the VVD mutants. We saw significantly reduced interaction between WCC and VVD in the fastest photocycle mutant when compared to the WT strain and the other mutants (**Fig 3C and 3D**). We also routinely saw slightly increased VVD-WCC interaction in the slowest photocycle mutant; however, this difference was not significant unlike the difference in VVD-WCC interaction between the fastest photocycle mutant and the WT strain (**Fig 3D**). These results strongly suggested it was the reduced VVD-WCC interaction in the fastest photocycle mutant that was responsible for the partial loss of photoadaptation.

Reduced VVD-WCC interaction has been previously shown to be involved in photoadaptation defects in two well described VVD mutants (*vvdC71S* and *vvdC108A*) [25,43]. However, these mutants do not have altered photocycle kinetics *in vitro* thus making the *vvdI74VI85V* mutant unique. The reduced VVD-WCC interaction in turn allows for a greater fraction of the WCC to be activated by a light pulse even in the presence of the mutant VVD protein in the fastest photocycle mutant (**Fig 3B**). Thus the interaction between the proteins can be modulated simply by altering the photocycle of one.

### WC-1 stability is reduced in the fastest photocycle mutant even in the presence of FRQ as a second stabilizing factor

**Fig 4:**
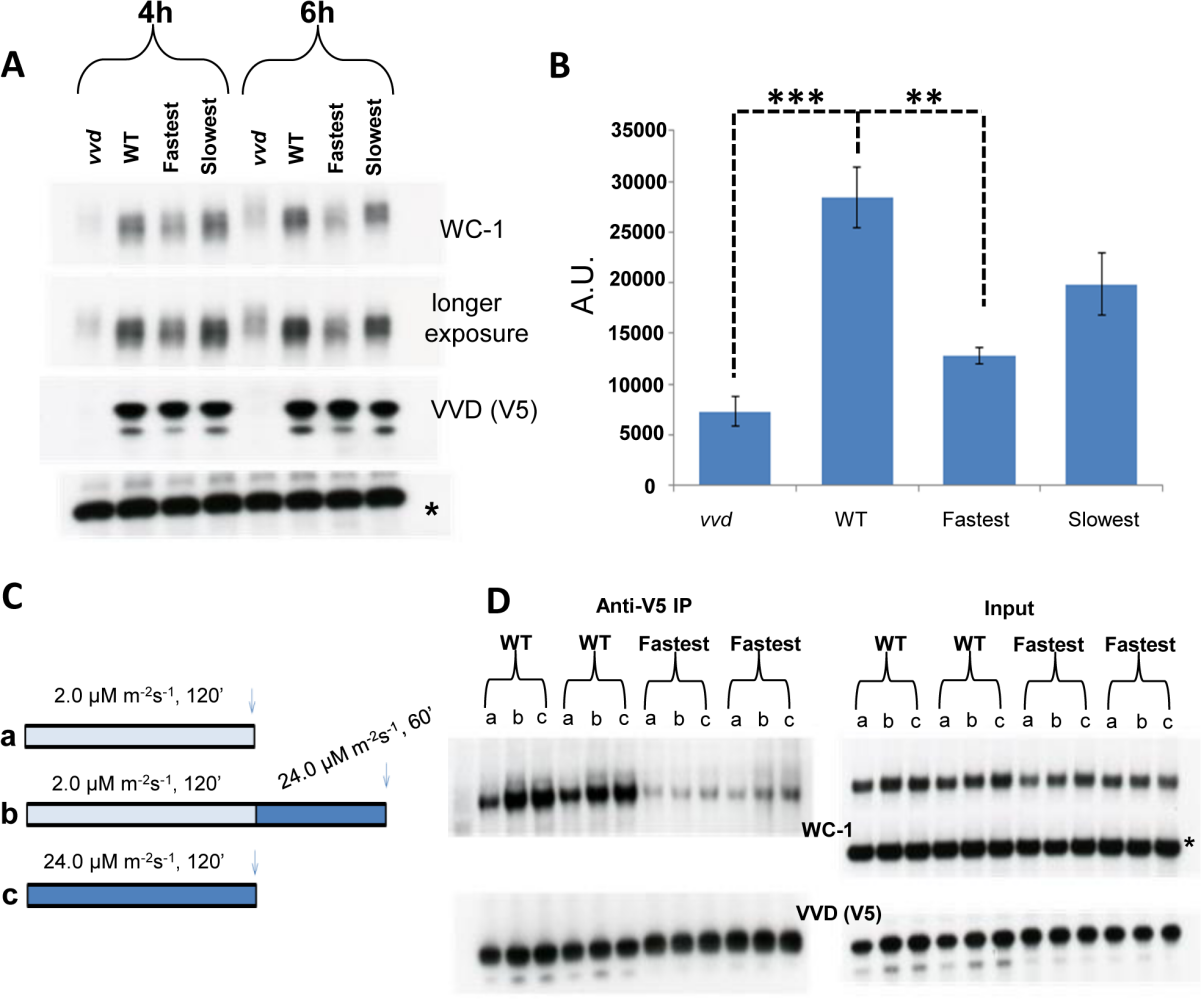
Reduced VVD-WCC interaction influences WC-1 stability in the fastest photocycle mutant and the system’s ability to respond to changing light intensity. (A) Strains were exposed to constant blue light (~30 μM m^-2^s^-1^) for 4 and 6 hours. Protein samples were harvested in the presence of phosphatase inhibitors and WC-1 stability was examined using Western blotting techniques. 6% Tris-Glycine gels were used instead of 3–8% Tris-Acetate gels for greater resolution. (B) 3 blots from the 6 hour time point were quantified via densitometry using NIH Image J software. (C) Experimental design to examine the WT and fastest photocycle strains response to an increase in light intensity. Strains were either exposed to low light (blue) intensity (a), high light (blue) intensity (c) or low light intensity (blue) followed by exposure to high light (blue) intensity (b). (D) Co-IP experiments (duplicate biological replicates shown) were performed after cross linking as previously described in Fig 3 legend to examine VVD-WCC interaction after light treatments shown in (C); a,b and c on the blots correspond to conditions in (C) * non specific band /loading control. Asterisks indicate statistical significance when compared with WT as determined by an unpaired *t* test. ***P<0.001, **P<0.01.

During the process of photoadaptation, the interaction between VVD and WCC has been reported to play a role in stabilizing light-activated WC-1 [19,35]. In constant light levels of WC-1 are reduced in a *vvd* null strain when compared to a wild-type strain, suggesting possible increased WC-1 turnover in the absence of VVD. The core clock protein FRQ (FREQUENCY) also plays a role in stabilizing both dark and light-activated WC-1 independent of VVD [44–46]. WC-1 activates the transcription of the *frq* gene and after translation FRQ physically interacts with WC-1, inhibits WC-1 transcriptional activity in the dark and promotes the accumulation of WC-1 [47–50]. Because the fastest photocycle mutant showed reduced VVD-WCC interaction we asked if this reduced interaction influences WC-1 stability in a background where FRQ is present. To test this, we grew mycelia from our strains in the dark for 48 hours, exposed them to bright light (~30 μM m^-2^s^-1^) for 4 and 6 hours, using blue light to avoid photoreversion, and then isolated protein in the presence of phosphatase inhibitors. As expected, WC-1 was hyperphosphorylated compared to WT and its levels were constantly low at both exposure times in the *vvd* null strain (**Fig 4A**). Tellingly, we saw statistically significantly reduced WC-1 levels in the fastest photocycle mutant compared to the WT and slowest strains at both the 4 and 6 hour time points, indicating that the extent of interaction between VVD and WCC in this strain is influencing the level of WC-1 even in the presence of FRQ as an independent stabilizing factor (**Fig 4A and 4B**).

Transcriptionally active WC-1 is associated with phosphorylation and subsequent degradation [44,51]. In Figs 1D and 3B we show WCC to have enhanced activity in the fastest photocycle mutant. Our stability data (**Fig 4A and 4B**) suggests that under the condition of reduced VVD-WCC interaction not only is a greater portion of WC-1 transcriptionally active but the post-transcriptional accumulation of WC-1 may also be reduced, possibly because light-induced, newly synthesized WC-1 is more prone to being transcriptionally active and destined for degradation in the absence of appropriate levels of interaction with the stabilizing factor VVD.

### The fastest photocycle mutant fails to establish a “dynamic” VVD-WCC pool that can respond to changing light intensity

VVD has also been described as playing a role in maintaining sensitivity to changes in light intensity during daytime [19,35,52]. We sought to understand biochemically how the VVD-WCC interaction is influenced in the WT strain and the fastest photocycle mutant when the strains are exposed to two different light intensities, or when they are exposed to a higher light intensity after being photoadapted at a lower light intensity. The strains were exposed to either (a) 2 μM m^-2^s^-1^ or (c) 24 μM m^-2^s^-1^ of blue light for 2 hours or (b) 2 μM m^-2^s^-1^ of blue light for 2 hours then exposed to 24 μM m^-2^s^-1^ of blue light for 1 hour (**Fig 4C**) followed by DSP cross-linking and Co-IP analysis (**Fig 4D**). In the WT strain more WC-1 was pulled down with V5 tagged VVD after a 2 hour exposure to the higher light intensity when compared to a 2 hour exposure at the lower light intensity (**Fig 4D**) due to a combination of more WC-1 synthesis and the presence of more light-activated WC-1 at the higher light intensity [35]. Importantly these data are not complicated by an unmeasured accumulation of light-reverted VVD due to the presence of white light. After an initial low light exposure for 2 hours, the WT strain is able to respond to an increase in light intensity to 24 μM m^-2^s^-1^ and attain a new VVD-WCC interaction state as seen by increased VVD-WCC interaction. This is in stark contrast to the VVD-WCC pool in the fastest photocycle mutant which appears to maintain a largely equivalent, quite low level of interaction under all light intensities including when the light intensity is changed from low to high (**Fig 4D**). This is due to a combination of reduced WC-1 stability in this mutant (**Fig 4A**) and the inherently reduced VVD-WCC interaction that makes mutant VVD unable to interact with WC-1 even at higher light intensity with more WC-1 available. Thus, accelerating the photocycle causes reduced VVD-WCC interaction and leads to an abolition of responsiveness to changing light-intensity, thus hampering the establishment of a dynamic VVD-WCC pool.

### *frq* transcription is responsive to changing light intensity in the WT but not in the fastest photocycle mutant

**Fig 5:**
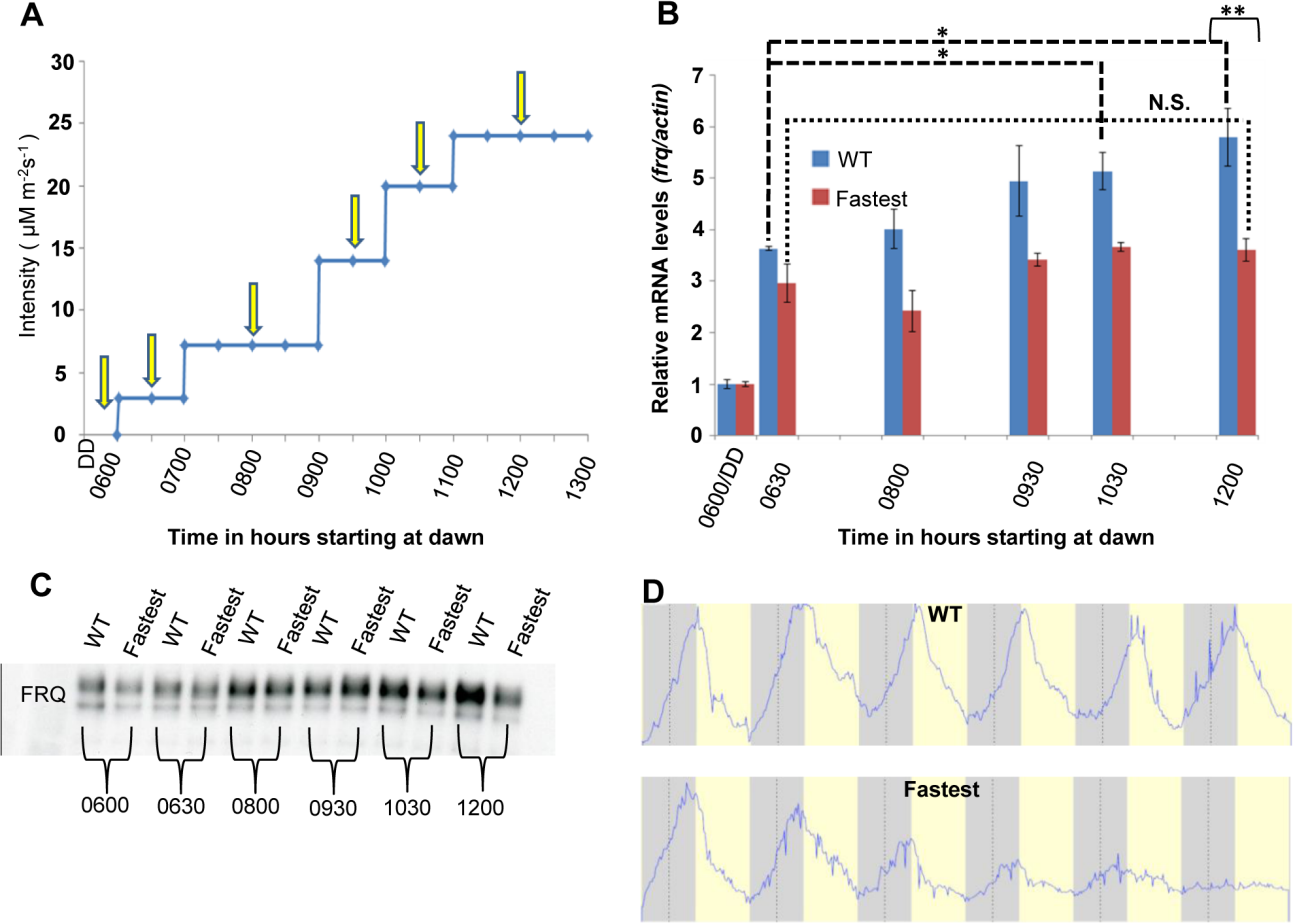
The WT strain increases *frq* transcription in response to increasing light intensity whereas the fastest photocycle mutant does not and exhibits marked circadian defects in quasi-normal LD cycles. (A) Experimental design to examine *frq* mRNA and FRQ protein levels in response to increases in light. Yellow arrows indicate times of harvest. Blue light intensity was increased using an externally controlled LED incubator to mimic increasing blue light intensity during the first half of a daily light cycle. (B) *frq* mRNA levels (n = 3 for each strain) in WT(blue) and fastest photocycle mutant strain (red) was determined using RT-PCR at time points shown in panel (A). (C) Western blot showing FRQ protein levels in the WT and fastest photocycle mutant strain at time points corresponding to panel (A). (D) Averaged densitometric traces of race tubes for WT (n = 2) and the fastest photocycle mutant (n = 4) grown under quasi-normal 12 hour light: 12 hour dark (LD) cycles with maximum light intensity limited to ~10 μM m^-2^s^-1^. Asterisks indicate statistical significance as determined by an unpaired *t* test. ***P<0.001, **P<0.01, *P<0.05, N.S. difference not significant.

We have shown biochemically how sensitivity to increasing light intensity might be achieved through a dynamic VVD-WCC pool (**Fig 4D**) so we followed how the system responds to increasing light intensities at the transcriptional level. The context we used was the clock gene *frq* as little is known about how the clock responds to changing light intensity during day-light hours. Using an externally controlled blue light LED panel we mimicked increasing light intensity during the first half (6 hours) of a 12 hour light: 12 hour dark cycle and assayed *frq* mRNA and protein levels under these conditions. The WT strain is responsive to increasing light intensity as seen by the increase in *frq* mRNA levels resulting from several step increases in fluence levels (**Fig 5B**). In contrast, after an initial response to the first light treatment after darkness similar to the WT strain (0630 time point in Fig 5B), the fastest photocycle mutant shows no significant increase in *frq* transcript in response to increases in light intensities. The difference in WT *frq* amounts is statistically significant at the 12:00 hour sampling point and we also see significantly higher FRQ protein levels in the WT strain when compared to the fastest photocycle mutant at this time (**Fig 5B and 5C**). We interpret these data to indicate that as light intensity is increased, both auto-regulatory WC-1 and VVD are increased such that each step yields a greater pool of VVD-WCC in combination with that carried over from the previous lower light intensity; in contrast, the fastest photocycle mutant pool is unable to accumulate so the response is always similar to that seen upon dark to light. In WT under white light, unlike in the fastest photocycle mutant, more WCC from the VVD-WCC pool is being made available to respond to each new light intensity step (through the combination of photoreversion and transient dissociation, **see model**) following which a new VVD-WCC equilibrium pool is established. In contrast, the fastest photocycle mutant has reduced WC-1 stability combined with the inability to respond to changing light intensity and this translates into an aberrant *frq* mRNA and protein profile. In addition, we were unable to detect VVD (WT and mutant) at the *frq* locus using chromatin-immunoprecipitation, suggesting that the VVD-WCC pool might be the main functional unit for maintaining sensitivity to increasing light (**S3 Fig**).

### The fastest photocycle mutant shows marked defects in circadian output under quasi-normal light-dark cycles

Apart from photoadaptation, VVD plays an important role in the circadian system. Although not required for rhythmicity in constant conditions *per se*, VVD influences the turnover of *frq* RNA and impacts phase setting [26] at the light-to-dark (dusk) transition [38]. A strain that lacks VVD shows a ~4 hour phase delay and is unable to take cues from the photoperiodic history preceding the point of lights off [38]. We studied the dynamics of *frq* mRNA decay after a light (20 h bright white light)-to-dark transition in our photocycle mutant strains to see if *frq* mRNA turnover is altered in these mutants. As expected, the *vvd* null strain showed a delay in *frq* mRNA turnover when compared to the WT strain (**S4A Fig**). However, the fastest and the slowest photocycle mutants did not show any significant difference in bulk *frq* mRNA decay when compared to WT. This was not due to a difference in turnover rates of VVD itself (that might have resulted from the mutations introduced) (**S4B and S4C Fig**), ruling out the possibility that the fastest photocycle variant is more stable. Rather, the delayed disappearance of *frq* mRNA in the *vvd* null strain is thought to reflect sustained WCC transcriptional activity after the light-to-dark transfer, and this extended period of elevated *frq* mRNA explains the observed phase delay [38]. These data suggest that although the fastest photocycle mutant shows reduced VVD-WCC interaction, the interaction that persists is apparently sufficient to maintain a normal *frq* mRNA decline in this strain following dusk. Reduced overall WC-1 activity in the fastest photocycle mutant, because of decreased WC-1 stability and degradation on constant light exposure, could also help compensate reduced VVD-WCC interaction at the light-to-dark transition. To confirm that there are no circadian phase defects under constant conditions associated with our photocycle mutants we crossed the strain into a *ras-1* ^*bd*^ background [53] and repeated the light-to-dark synchronization experiment in a growth (race) tube. The *ras-1* ^*bd*^ mutation is a mildly activating point mutation of *ras-1* that makes it easier to observe rhythmic circadian-driven asexual spore formation when it is present in the genetic background. As we predicted, the fastest photocycle mutant did not show any kind of phase delay when compared to the WT while the *vvd* null strain presented the previously described approximately 4h phase delay (**S5A and S5B Fig,** [26]). The photocycle length of VVD does not appear to play a role in phase determination of the circadian system at least under constant (free-running) conditions.

It has been previously reported using “artificial moonlight conditions” (0.24 μM m^-2^s^-1^) that VVD plays a role in attenuating light-resetting by low light during night. In the previous study, exposing the *vvd* null strain to this low light intensity was reported to lead to progressive loss of rhythmicity (after day 3–4) whereas the WT showed persistent rhythms [35]. We were, however, unable to replicate these results (**S6A Fig**). Moonlight is typically 0.2 lux, up to 1 lux on mountaintops with clear air [e.g. http://en.wikipedia.org/wiki/Moonlight,[54]], the equivalent of 0.002–0.01 micro moles photons m^-2^ sec^-1^ for the cool white fluorescent lights used here (e.g. http://www.apogeeinstruments.com/conversion-ppf-to-lux; http://en.wikipedia.org/wiki/Talk%3ALumen_%28unit%29). We found that the *vvd* null strain maintains rhythmicity beyond 3–4 days under bright artificial moonlight conditions (0.02 μM m^-2^s^-1^) and does so at light levels even tenfold higher than natural moonlight (0.2 μM m^-2^s^-1^) (**S6B Fig**).

**Fig 6:**
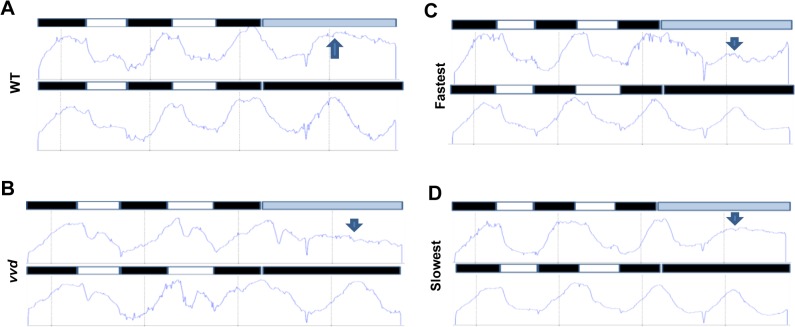
Photochemical alterations in the fastest photocycle mutant affect the circadian clock during the day phase of an LD cycle. Densitometric traces of WT (A), *vvd* null (B), fastest (C) and the slowest (D) photocycle mutant (race tube images in **S8 Fig**). Traces are an average of 3–6 race tubes. The strains were entrained to 12:12 LD cycles for 2 days using blue light (30 μM m^-2^s^-1^) followed by release into either constant low blue light (~2 μM m^-2^s^-1^) or constant dark. Arrows in blue show the expected phase of peak conidiation during the first day in constant light as determined by phase of the peak during the first day in constant dark. The WT and the slowest mutant strains maintain the expected phase of conidiation whereas the *vvd* and the fastest mutant strain do not.

VVD plays a role in maintaining the circadian clock in the light phase of the cycle and the main function of VVD is to prevent clock resetting at dawn [38]. This implies a role for VVD during the day phase of a light:dark (L:D) cycle and we have shown that photocycle length does affect *frq* mRNA levels under a quasi-normal daytime increase in light intensity (**Fig 5B**). Therefore we tested whether the fastest photocycle mutant has an associated circadian phenotype under increasing light intensity conditions. Strains were grown on a 12:12 light:dark (L:D) cycle in which the light (blue wavelength) intensity changed during the day phase from low (dawn) to high (mid-day/noon) and back to low (dusk) but with a maximum intensity of 10 μM m^-2^s^-1^ (**Figs 5D and S7A**). Under these conditions circadian output in the fastest photocycle mutant dampened initially and became arrhythmic. We saw similar dampening of rhythms in the *vvd* null strain under these conditions (**S7A Fig**) but no complete arrhythmicity suggesting that the *vvd* null strain might perceive the light phase of the LD cycle as a complete resetting cue. This is most likely due to the *vvd* null being phase-locked to dusk irrespective of the photoperiodic history. It has also been shown that the clock is broken (constantly reset) in the light phase of full photoperiods in the *vvd* null strain [38], a result that we have replicated here (more details below, **Fig 6**). In contrast, we think that the fastest mutant does not completely reset like the *vvd* null strain in the light phase but has aberrant phase resetting (due to partially functional VVD) which is carried over to the next LD cycle. Similar dampening and phase defects were seen in the fastest photocycle mutant when the maximum light intensity was 2.4 fold higher (~24 μM m^-2^s^-1^) although fewer strains became arrhythmic (**S7B Fig**). Biochemical dissection of this phenotype is difficult as the strains start losing rhythmic conidiation only after day 4 or 5 thereby precluding protein and mRNA analysis in liquid cultures; however, the data shown in Figs 4A and 5B indicate that the fastest photocycle mutant has an aberrant response to light (reduced WC-1 stability and aberrant *frq* transcription) and these defects may accumulate over 3–4 days leading to defects in phase and rhythmic conidiation.

To further confirm that the photocycle alterations in the fastest photocycle mutant were affecting the clock during the day phase of an LD cycle the strains were entrained for 2 days to 12:12 L:D cycles using ~30 μM m^-2^s^-1^ blue light followed by release into either constant darkness (DD) or low intensity blue light (~2μM m^-2^s^-1^, LL) **(Fig 6)**. If the VVD in the system is able to prevent clock resetting and maintain the clock after release into constant light then we expect to see an overlap between the first conidiation peaks in LL and DD as has been previously described [38]. It can be clearly seen that the first conidiation peak in LL and DD overlap in the case of the WT and the slowest photocycle mutant (**Figs 6A, 6D and S8**). However, this is not the case for the *vvd* null strain and the fastest photocycle mutant. In the fastest photocycle mutant we see a much reduced peak in constant light (compare peak height of LL peak with preceding peak) (**Fig 6B and 6C**). This strongly suggests that photochemical alterations in the fastest photocycle mutant affect VVD function and its ability to maintain the clock during the day phase of full photoperiods.

## Discussion

Although *in vivo* studies have probed the biology of photoreceptors utilizing null or blind mutants [5,8,55], and *in vitro* physicochemical studies have framed questions and raised hypotheses [3], the functional relevance and adaptive significance of the conservation of a wide range of photocycle lengths in different photoreceptors has not before been tested experimentally. Good context for this effort, however, is provided by studies showing that thermal reversion rates are genetically manipulable in photoreceptors including both LOV domain proteins such as VVD [20] and in phytochrome [56,57]. For instance, mutations surrounding the bilin binding pocket in PhyB can speed or slow the rate of thermal reversion as well as independently impacting photochemistry and nuclear localization patterns [56]. These data establish the mutability of these characteristics, but interestingly there is no evidence for natural variation in photocycle length of phytochromes suggesting that evolutionary selection has not tuned the kinetics of photo-adduct decay in phytochromes to specific tasks as it has for LOV domains in which natural variation has provided a rich repertoire of orthologous photoreceptors whose dark (thermal) reversion rates vary over several log orders. Given this natural variation the LOV domain photoreceptors seemed an excellent framework in which to probe the biological significance of photocycle length, and the tractable photobiological and circadian model *N*. *crassa* provided the perfect context for studying photocycle mutants. We have shown here that reducing the photocycle length of the blue light photoreceptor VVD has dramatic consequences on light and circadian biology that are phenotypically separable from the null phenotype. While current models of VVD function emphasize the formation of a VVD-WCC pool that provides light- activatable WCC through thermal reversion of the VVD-WCC heterodimer [25,35,36,58], these models ignore the contribution of near UV-driven photoreversion of both VVD and WC-1 photoreceptors. By considering and controlling this variable, and by tuning the magnitude of the photoreversion-generated VVD-WCC pool using defined *in vitro* guided VVD mutations, we show how a dynamic VVD-WCC pool is important for VVD function, and link VVD photochemistry with the size of this pool. However, it is important to note that despite the phenotype and its clear dissection using molecular and biochemical techniques, we cannot confirm with absolute certainty that the mutant proteins retain the *in vitro* photocycle characteristics in an *in vivo* setting.

The VVD-WCC interaction is transient [25,35] suggesting that WCC from the VVD-WCC pool can be made available for transcription. Even in the photo-adapted state a fraction of WCC is required to be transcriptionally active, because blocking total protein synthesis including that of VVD, leads to loss of photoadaptation [19]. This active fraction of WCC can induce the synthesis of WC-1 [59,60] and hence replenish the fraction lost through transcription-induced degradation [44,50]. The second source of activated or light-activatable WC-1 is through thermal reversion of the activated VVD, the VVD photocycle that has been examined here. The third source of light-activatable WC-1 is through the process of near-UV stimulated photoreversion [2] which generates dark VVD and WC-1 that can then be light-activated to drive expression of more WC-1 and VVD. Thus, WC-1 is the primary sensor of light intensity in the system; light drives WC-1 and VVD expression, activates them to heterodimerize, and also stimulates the photoreversion of the complex. So long as a single WC-1 results in synthesis of more than just one WC-1 and one VVD, the size of the protected VVD-WCC pool must increase with light intensity. The magnitude of the VVD-WCC pool plays a pivotal role in determining the amount of WCC available for transcription as well as the amount of light-activated WC-1 available for reversion to the dark form.

In this study we have shown that a double mutation (I74VI85V) that dramatically reduces the photocycle length of VVD *in vitro* [20] has a profound effect on VVD biological function, whereas another mutation (M135IM165I) [20] that dramatically lengthens the photocycle appears essentially wild type in the assays we have used. This suggests that under normal light conditions photo-reversion dominates thermal reversion in determining the ratio of the light to dark forms [3]. In VVD, I74VI85V increases the light to dark thermal reversion rate (decreased photocycle length); this accelerated thermal reversion comes to dominate the rate of photoreversion and affects the steady state ratio of the light:dark form (53:47 in mutant vs. 92:8 in WT) [20]. The result is that this fastest photocycle mutant shows a partial loss of photoadaptation which we confirmed at the level of gene expression (**Fig 1B and 1D**). VVD physically interacts with WCC to attenuate its transcriptional activity [25,35,36] and a 2 pulse experiment and subsequent protein cross-linking assay showed that this interaction is compromised in the fastest photocycle mutant (**Fig 3**). The reduced interaction in the fastest mutant strongly supports the previous, unproven hypothesis that it is indeed light-activated VVD that is functionally active [25,35,36,58]. From the signaling point of view, light-activation via photon absorption leads to flavin adduct formation at the active cysteine which transduces a conformational change in VVD protein, and this light-activated structure is required for the VVD-WCC interaction. It has been shown that dissociating VVD photochemistry from structural change (e.g. *vvdC71S*) leads to a functionally dead VVD protein [16]. Thus, we attribute the reduced VVD-WCC interaction in the fastest photocycle mutant to the greater rate of light-state-to-dark state reversion, which contributes to the reduced steady state ratio of the light:dark form, thus lowering the amount of functionally active light form of VVD available at any light intensity.

We examined the consequences of reduced VVD-WCC interaction on the *N*. *crassa* light and circadian systems. WC-1 is highly unstable in the absence of VVD [19,35] presumably because more WC-1 is available for transcription and WC-1 transcriptional activity is linked to its degradation [44,45,50,61]. Consistent with this WC-1 is less stable in the fastest photocycle mutant when compared to WT on prolonged light exposure (**Fig 4A and 4B**). Interestingly, WC-1 stability in the fastest photocycle mutant lies between that of WT and the *vvd* null strain suggesting that the strength of the VVD-WCC interaction is directly correlated with WC-1 stability. This indicates that although WCC is more active in the fastest photocycle mutant, a greater fraction of the newly synthesized WC-1 is available for transcription and hence more prone to degradation.

The reduced VVD-WCC interaction in the fastest mutant also leads to an aberrant response to changing light intensity. The VVD-WCC pool is sensitive to increasing light intensity in WT but not in the fastest photocycle mutant (**Fig 4C and 4D**) where reduced VVD-WCC interaction results in a smaller less dynamic VVD-WCC pool. The VVD-WCC pool in turn determines the amount of WC-1 available for transcription and photoreversion, so both these factors determine how the system will respond to the next higher light intensity. Light is a major environmental cue for circadian clocks and light input into the *N*. *crassa* clock is gated through WCC–mediated transcription of *frq* [47,52,62]. Indeed *frq* expression increases with increasing light intensities in WT but not in the fastest mutant (**Fig 5B**), consistent with the finding that WC-1 is less stable and the smaller VVD-WCC pool cannot respond to increases in light intensity in this strain. The adaptive significance of pool size and photocycle length was revealed by showing that the fastest photocycle mutant loses overt rhythmicity when grown under a quasi-normal LD cycle with steps up to and down from a maximum intensity of 10 μM s^-1^m^-2^ approximating a very overcast day (**Fig 5D**). Additionally, release experiments show that the photochemical alterations in the fastest photocycle mutant affect its function in preventing resetting at dawn and maintaining the clock in the day phase (**Fig 6**). The interpretation is that the normal extended photocycle length is needed to maintain the oscillator and its phase information during the day, as well as to allow the pool of VVD-WCC to grow with light intensity to keep *frq* transcription and FRQ levels high enough that the dark-initiated decay of FRQ marks a clear transition. The combination of the two functions maintains robust cycling over several days.

The dominance of the thermal reversion rate (K_t_) [3] over photoreversion in the *vvdI74VI85V* mutant can also help describe the loss of overt rhythmicity under our quasi-normal LD cycle. It has been modeled that K_t_ determines the light-to-dark ratio as a function of the fluence [3]. The K_t_ of the photoreceptor ZTL in *Arabidopsis thaliana* is 1.6x10^-4^ and that of wild-type VVD is 5.6x10^-5^. Interestingly the fastest photocycle VVD has a K_t_ >>ZTL (3.5x10^-2^)[20]. This model places the WT VVD in a class of photoreceptors where light:dark ratio is skewed completely towards the light state even at very low light intensities. However, this model also places the fastest photocycle VVD in a class where its light:dark ratio is sensitive to low light intensity, which would imply that a very small fraction of the molecules are in the light state under the conditions used in our step increase, quasi-normal light-dark cycles where the maximum light intensity was limited to 10 μM m^-2^s^-1^. This could potentially hamper the function of VVD[I74VI85V] at low light intensity, and hence the clock.

**Fig 7:**
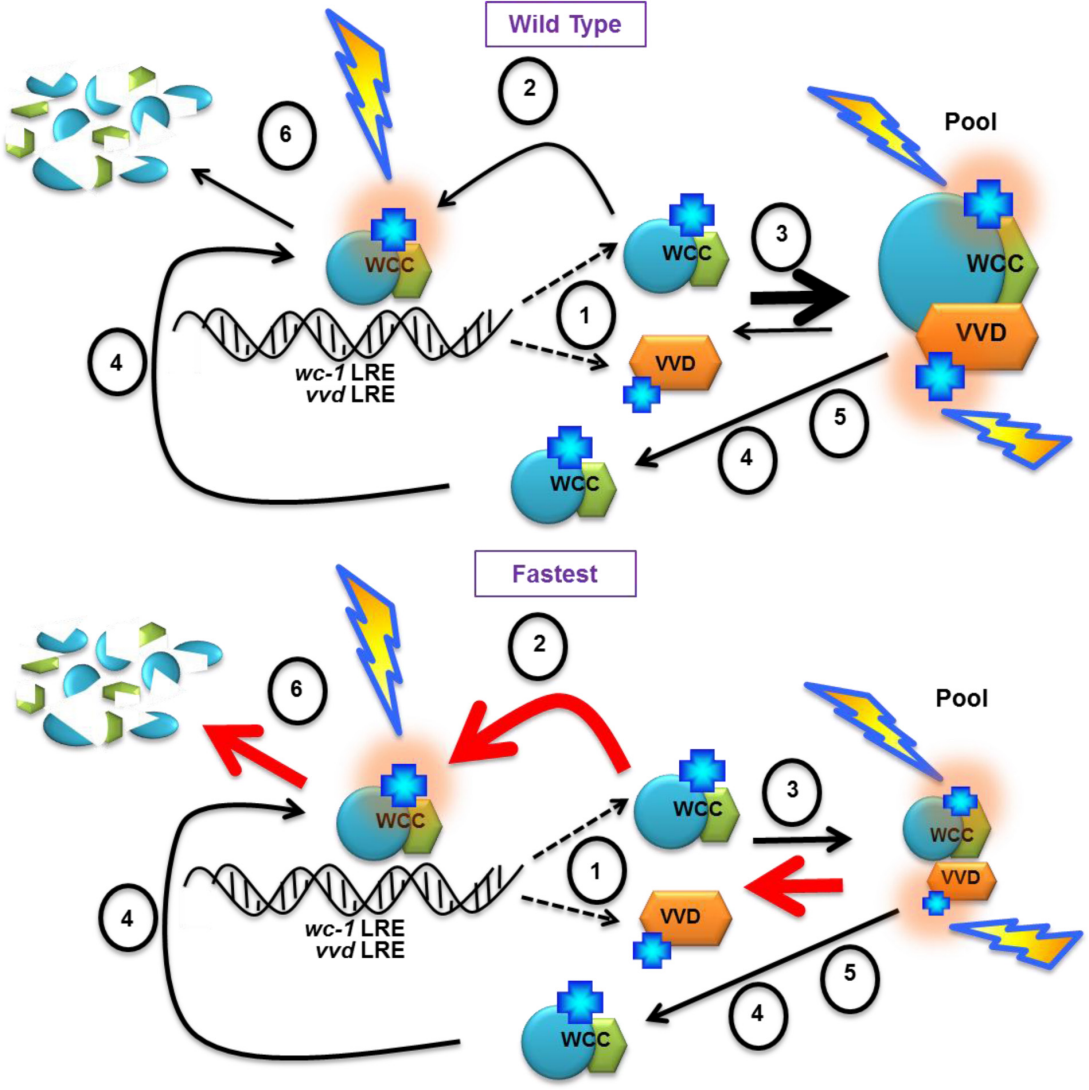
Model: Photocycle length defines the strength of the VVD-WCC interaction and the magnitude of this interacting pool is essential for photoadaptation and maintained light sensitivity. (1) WCC binds DNA light responsive elements (LRE) after light exposure, and activates the transcription of *vvd* and *wc*-1. Light activated chromophores (blue crosses) are shown with orange halo. A portion of WCC is subsequently degraded (6). Newly synthesized WC-1 protein may be light-activated to become part of transcriptionally active WCC (2) or complex with newly synthesized light-activated VVD forming a dynamic pool (3). (4) Ambient light intensity results in some portion of WC-1 undergoing photoreversion to the dark state, allowing future re-activation by light. (5) The length of the VVD photocycle controls the duration of the WCC-VVD interaction and the size of the WCC-VVD pool. This in turn determines how much stabile, light-activated WCC is maintained away from DNA and also available via photoreversion to dark WC-1. Thicknesses of arrows represent relative rates of reactions. Red arrows in the fastest photocycle mutant show rates controlled by increasing rate of adduct decay. See text for more details

Based on these data we propose a model where the native VVD photocycle length is required for the establishment of the dynamic VVD-WCC pool essential for photoadaptation and maintaining sensitivity to increasing light intensity. Light-activated transcriptionally active WC-1 in the WCC binds DNA light responsive elements (LRE) and activates the transcription of VVD and WC-1 [**Fig 7,(1)**]. Newly synthesized WC-1 can be light-activated adding to transcriptionally active WCC [**Fig 7, (2)**] or complex with newly synthesized light-activated VVD forming a dynamic pool [**Fig 7, (3)**]. A fraction of WC-1 determined by ambient light intensity also undergoes photoreversion in light and becomes available for another round of light activation [**Fig 7, (4)**]. The VVD photocycle length determines the duration of the VVD-WCC interaction and this in turn determines the magnitude of the pool of stabilized, light-activated WCC that spends time away from DNA and is available for photoreversion to dark WC-1 [**Fig 7, (5)**]. The reduced duration of the VVD-WCC interaction in the fastest photocycle mutant (**bottom**) increases the amount of WCC available for transcription (and subsequent degradation [**Fig 7, (6)**]) but also reduces the pool of light-activated WC-1 that is available for regeneration of dark WC-1 through photoreversion. This reduced pool size and lack of growth of the VVD-WCC pool with increasing light intensity are seen as defects in photoadaptation, both repression of WCC transcription and the inability to maintain sensitivity to changing light intensities.

## Materials and Methods

### *Neurospora crassa* strains and culture conditions

The *vvd* null strain was generated as part of the *Neurospora* knockout project in which the complete replacement of the gene with the selectable marker *hph* was verified by Southern analysis. The DNA constructs (containing 3.5kbp of the *vvd* promoter) encoding the tagged version of the VVD wild-type and mutant proteins were targeted to the *csr-1* locus of a *vvd* null strain using a previously described transformation protocol [41]. The strains were confirmed as construct knock-ins at the *csr-1* locus using PCR analysis (**S9 Fig**) (Forward 5’-TAACGCCAGGGTTTTCCCAGTCACGACG-3’, Reverse 5’-GCGGATAACAATTTCACACAGGAAACAGC-3’) that helps detect the absence of the *csr-1* ORF. For the race tube experiments strains were crossed with a strain carrying the *ras-1[bd]* mutation [53]. Strains were maintained on slants containing solid growth media containing 1x Vogel’s and 1.5% sucrose. Race tube media contained 1xVogel, 0.01% Glucose and 0.17% Arginine. All RNA and protein experiments were carried out with fungal plugs grown in Bird medium containing 1.8% glucose using techniques previously described [25]. Briefly, fresh (~1 week old) conidia were inoculated in petri-plates containing 20mL Bird medium and allowed to grow in constant dark for 48 hours. Then, using an 8mm diameter cork borer, plugs were cut from the mycelial mat and the individual plugs were placed in 125mL flasks containing 50ml Bird medium. Light exposure experiments were performed after another 24 hours of culturing on a shaker in constant darkness at 25°C. After the experiment, the mycelia were collected using filtration and immediately frozen in liquid nitrogen and stored at -80°C until subsequent protein and RNA isolation. All white light treatments were carried out using a broad spectrum (400–700nm) cool fluorescent bulb (General Electric). Blue light treatments were performed in a temperature-controlled incubator with an externally controlled blue LED panel (E30LED, Percival Scientific, 450nm) using specified intensities.

### Protein preparation and RNA isolation

Protein lysates were prepared using methods described earlier [25]. Western blots were performed with 12ug of protein unless noted otherwise. RNA was isolated using TRIzol reagent (15596–026; Invitrogen) and cDNA synthesis was carried out using SuperScript III first strand synthesis kit (18080–051; Invitrogen) using 1.5ug of purified RNA. Real-time PCR was carried out with QuantiTect SYBR green RT-PCR kit (204243; Qiagen) in an ABI 7500 Fast system.

### Co-IP assay and DSP cross-linking

Protein cross linking was performed using 2mM DSP (D3669; Sigma) using a protocol previously described [25]. Co-IP was performed as follows: 1mg of total protein incubated with anti-V5 antibody-coated agarose beads (A7345; Sigma) overnight at 4°C followed by six washes with cold protein extraction buffer. Elution was performed by incubating the beads with 2x Western blot sample buffer at 65°C for 20 minutes. Samples to detect proteins were run on pre-made gels (Novex, Life Sciences) and transferred onto PVDF membranes. Polyclonal antibodies were used for FRQ (1:250), WC-1 (1:250) and WC-2 (1:5000) and commercial monoclonal V5 antibody (1:5000, Invitrogen) was used for VVD. Goat Anti-Rabbit HRP conjugate and Goat Anti-Mouse HRP conjugate (BioRad) were used a secondary antibodies. Membranes for anti-V5 IP were developed using SuperSignal West Femto Maximum Sensitivity Substrate (Thermo Scientific) and the signal was captured using X-ray film (GE Healthcare). Western Blots were quantified using NIH ImageJ software.

### Fluorescence microscopy

Conidia were inoculated directly into a flask with baffles containing Bird medium to ensure that the mycelia do not clump. After 24 hours of growth in dark the samples were exposed to 60 minutes of bright white light (LL60) following which 2mL of the loose mycelia were incubated in a 14mL Falcon tube with 4% Paraformaldehyde for 20 minutes. After 3 washes with 1xPBS a small fraction of the fixed mycelia was incubated with 1xHoechst followed by fluorescence microscopy.

## Supporting Information

S1 Fig (A) Western blot showing the equal levels of VVD expression in strains expressing GFP (and V5) tagged versions of VVD mutant after a 60 minute white light exposure(40 μM m^-2^s^-1^). (**B-D)** The partial loss of photoadaptation is conserved in other light response genes: Strains (n = 3) were exposed to 60 minutes of bright white light and RT-PCR analyses was performed to study the gene expression levels of *sub*-1,*cry* and *al*-1 for**P<0.01 *P<0.05.
(TIF)

S2 Fig Strain expressing GFP tagged fastest photocycle mutant VVD recapitulates the partial loss of photoadaptation phenotype. Strains (V5 and GFP tagged) were grown on solid minimal medium growth slants and exposed to constant bright white light (40 μM m^-2^s^-1^) for 4–5 days.
(TIF)

S3 Fig VVD does not localize to the DNA and primarily functions to maintain a VVD-WCC “pool”. Strains were grown in dark for 48 hours and then exposed to bright white light (40 μM m^-2^s^-1^) for 30 minutes. Chromatin immunoprecipitation experiment was carried out after formaldehyde cross-linking using anti-V5 antibody against V5 tagged VVD and WC-1 (as control). *frq* PLRE and *frq* clock box were checked for enrichment of VVD using RT-PCR and WC-1 served as the control. As can be seen VVD is not enriched over background and there is no difference between the WT and the mutant VVD.
(TIF)

S4 Fig *frq* mRNA and VVD protein turnover are not influenced by photocycle alterations. **(A)** *frq* mRNA turnover was studied by exposing the strains (n = 3) to constant white light (40 μM m^-2^s^-1^) for 20 hours followed by dark transfer and sample collection was performed at indicated times followed by RNA isolation and RT-PCR analyses. (**B&C**) **VVD turnover is not altered in the mutants**. VVD turnover and half-life were studied by exposing strains to constant white light (40 μM m^-2^s^-1^) for 20 hours followed by dark transfer. Western blots **(B)** were quantified (densitometry) using ImageJ to determine half-life **(C)**.
(TIF)

S5 Fig Photocycle alterations do not lead to circadian phase defects in a light to constant dark transfer circadian experiment. **(A)** Race tube assay confirming the absence of a phase defect in the fastest photocycle mutant in a light to dark synchronization experiment. Synchronization was carried out by exposing the strains to 20 hours of constant white light (40 μM m^-2^s^-1^) followed by dark transfer. **(B)** Densitometric analysis of conidiation rhythm shows that the fastest photocycle mutant does not show a phase defect whereas, the *vvd* strain shows the expected ~4hour phase delay. Traces are an average of 4–6 race tubes.
(TIF)

S6 Fig Artificial moonlight conditions do not influence circadian rhythmicity. **(A)** Under artificial moonlight conditions (0.02μM m^-2^s^-1^) both WT and *vvd* strain show sustained rhythmicity beyond 3–4 days. The race tubes were exposed to 12:12 L:D cycles where light intensity in the dark phase was kept at 0.02 μM m^-2^s^-1^ to mimic moonlight intensity. Densitometric traces are average of 3 race tubes (for each strain). **(B)** The WT and the mutant strains show sustained rhythmicity even when the artificial moonlight light intensity is increased to ~10 fold over environmental levels (0.2μM m^-2^s^-1^). Densitometric traces are averages of 6 race tubes per strain.
(TIF)

S7 Fig (A) Race tubes used for densitometry in Fig 5D showing 2 tubes of the WT strain and 2 tubes each of 2 progenies expressing the fastest photocycle VVD. The *vvd* strain also showed dampening of rhythms when the light (blue) intensity was varied during the day phase from low (dawn) to high (mid-day/noon) and back to low (dusk) but with a maximum intensity of 10 μM m^-2^s^-1^. **(B)** Same experimental setup as in (A) but with the maximum intensity of 24 μM m^-2^s^-1^.
(TIF)

S8 Fig Photochemical alterations in the fastest photocycle mutant affect VVD function and its ability to maintain the clock during the day phase of full photoperiods. Race tubes used for densitometry in **Fig 6.** Strains were entrained for 2 days 12:12 LD cycles using bright blue light (~30 μM m^-2^s^-1^) before being released in either constant darkness or constant low light intensity blue light (~2μM m^-2^s^-1^).
(TIF)

S9 Fig PCR genotyping to confirm integration of constructs at the *csr-1* locus. Actin was used as a control for amplification and primers to check integration were designed to sites flanking the site of integration.
(TIF)
