# Supplementary figures and images for: Biological Significance of Photoreceptor Photocycle Length: VIVID Photocycle Governs the Dynamic VIVID-White Collar Complex Pool Mediating Photo-adaptation and Response to Changes in Light Intensity
Source: PLoS Genet. 2015 May 15;11(5):e1005215. doi: 10.1371/journal.pgen.1005215 (PMC4433212; doi:10.1371/journal.pgen.1005215)

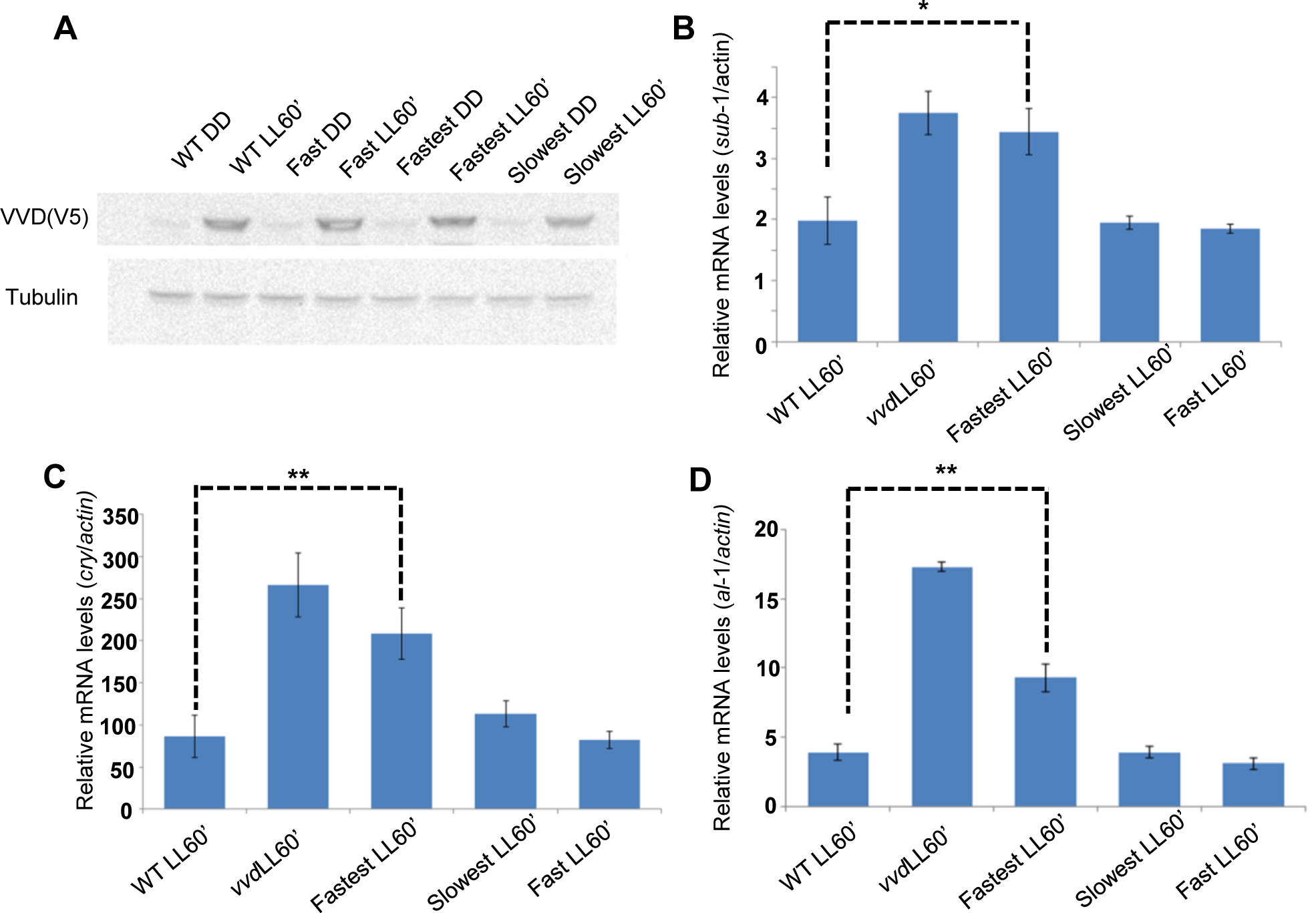

Supplement: S1 Fig — (B-D) The partial loss of photoadaptation is conserved in other light response genes: Strains (n = 3) were exposed to 60 minutes of bright white light and RT-PCR analyses was performed to study the gene expression levels of sub-1,cry and al-1 for**P<0.01 *P<0.05. (TIF) [file pgen.1005215.s001.tif]

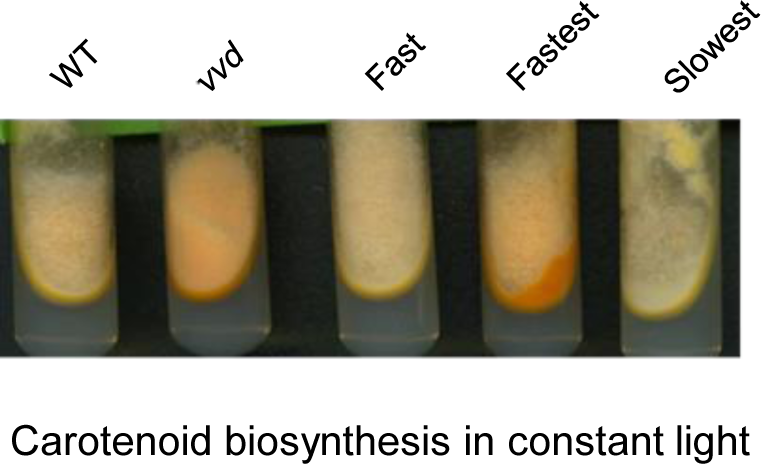

Supplement: S2 Fig — Strains (V5 and GFP tagged) were grown on solid minimal medium growth slants and exposed to constant bright white light (40 μM m-2s-1) for 4–5 days. (TIF) [file pgen.1005215.s002.tif]

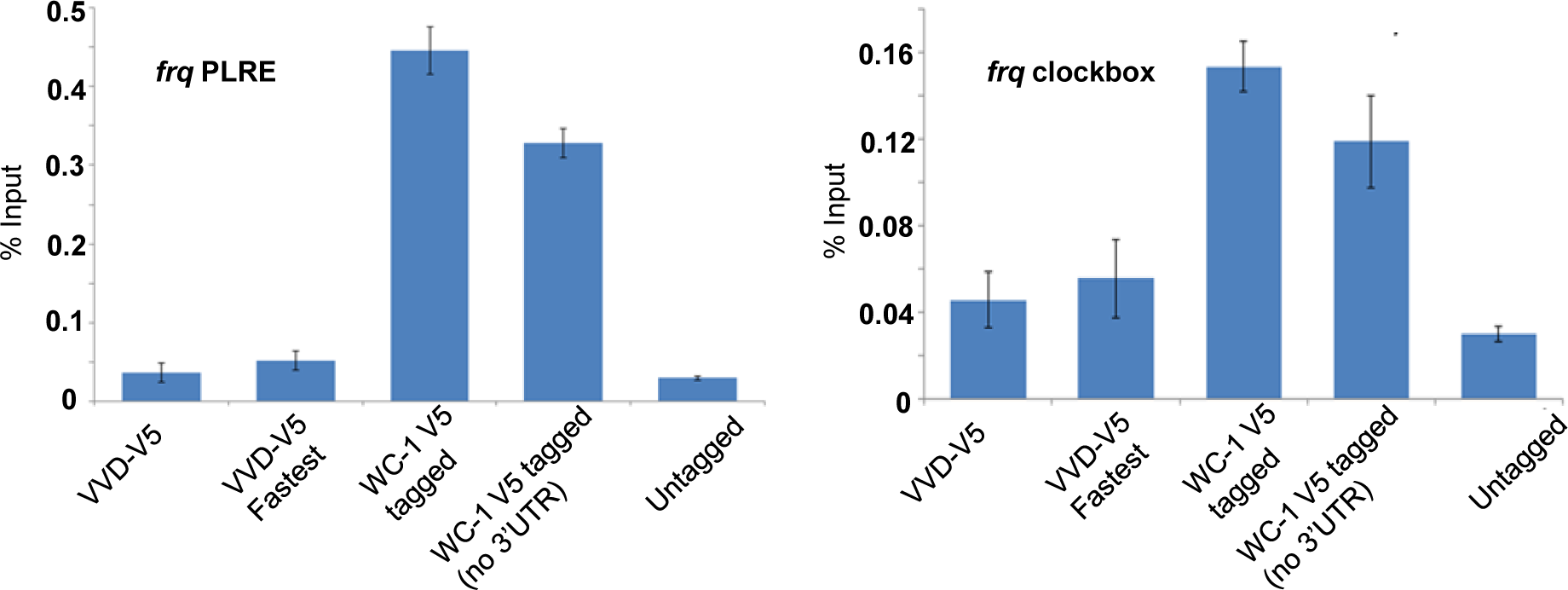

Supplement: S3 Fig — Strains were grown in dark for 48 hours and then exposed to bright white light (40 μM m-2s-1) for 30 minutes. Chromatin immunoprecipitation experiment was carried out after formaldehyde cross-linking using anti-V5 antibody against V5 tagged VVD and WC-1 (as control). frq PLRE and frq clock box were checked for enrichment of VVD using RT-PCR and WC-1 served as the control. As can be seen VVD is not enriched over background and there is no difference between the WT and the mutant VVD. (TIF) [file pgen.1005215.s003.tif]

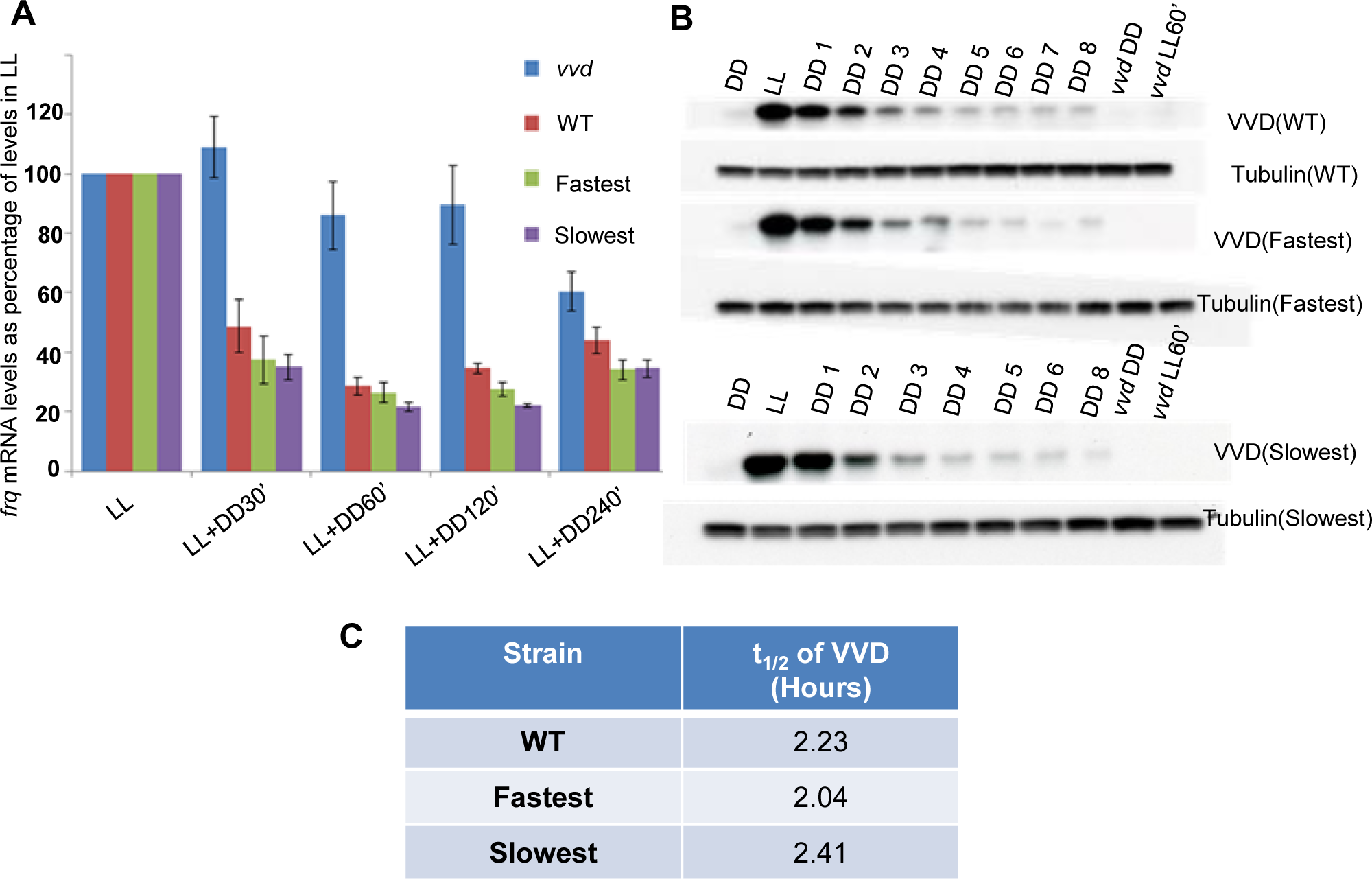

Supplement: S4 Fig — (A) frq mRNA turnover was studied by exposing the strains (n = 3) to constant white light (40 μM m-2s-1) for 20 hours followed by dark transfer and sample collection was performed at indicated times followed by RNA isolation and RT-PCR analyses. (B&C) VVD turnover is not altered in the mutants. VVD turnover and half-life were studied by exposing strains to constant white light (40 μM m-2s-1) for 20 hours followed by dark transfer. Western blots (B) were quantified (densitometry) using ImageJ to determine half-life (C). (TIF) [file pgen.1005215.s004.tif]

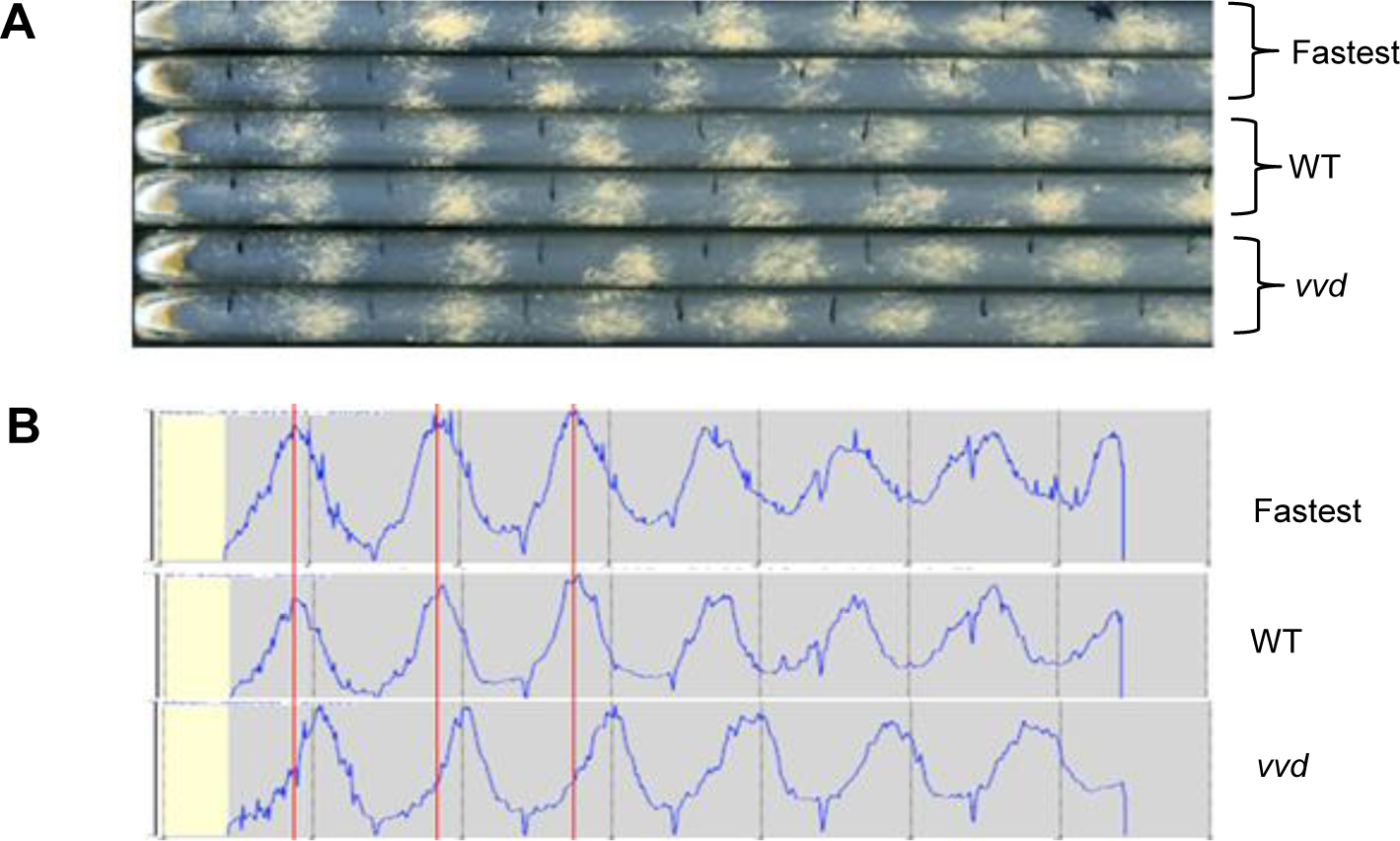

Supplement: S5 Fig — (A) Race tube assay confirming the absence of a phase defect in the fastest photocycle mutant in a light to dark synchronization experiment. Synchronization was carried out by exposing the strains to 20 hours of constant white light (40 μM m-2s-1) followed by dark transfer. (B) Densitometric analysis of conidiation rhythm shows that the fastest photocycle mutant does not show a phase defect whereas, the vvd strain shows the expected ~4hour phase delay. Traces are an average of 4–6 race tubes. (TIF) [file pgen.1005215.s005.tif]

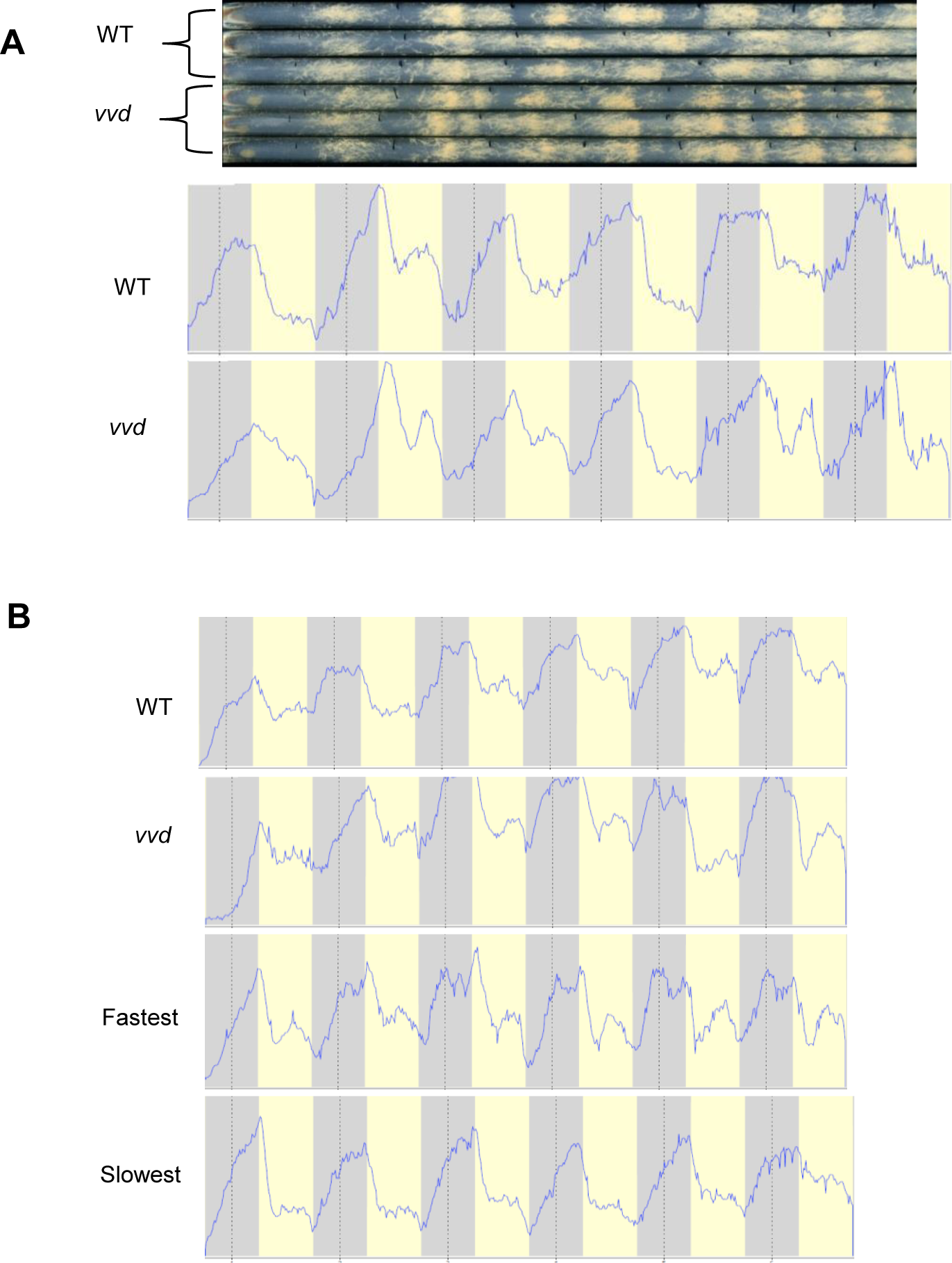

Supplement: S6 Fig — (A) Under artificial moonlight conditions (0.02μM m-2s-1) both WT and vvd strain show sustained rhythmicity beyond 3–4 days. The race tubes were exposed to 12:12 L:D cycles where light intensity in the dark phase was kept at 0.02 μM m-2s-1 to mimic moonlight intensity. Densitometric traces are average of 3 race tubes (for each strain). (B) The WT and the mutant strains show sustained rhythmicity even when the artificial moonlight light intensity is increased to ~10 fold over environmental levels (0.2μM m-2s-1). Densitometric traces are averages of 6 race tubes per strain. (TIF) [file pgen.1005215.s006.tif]

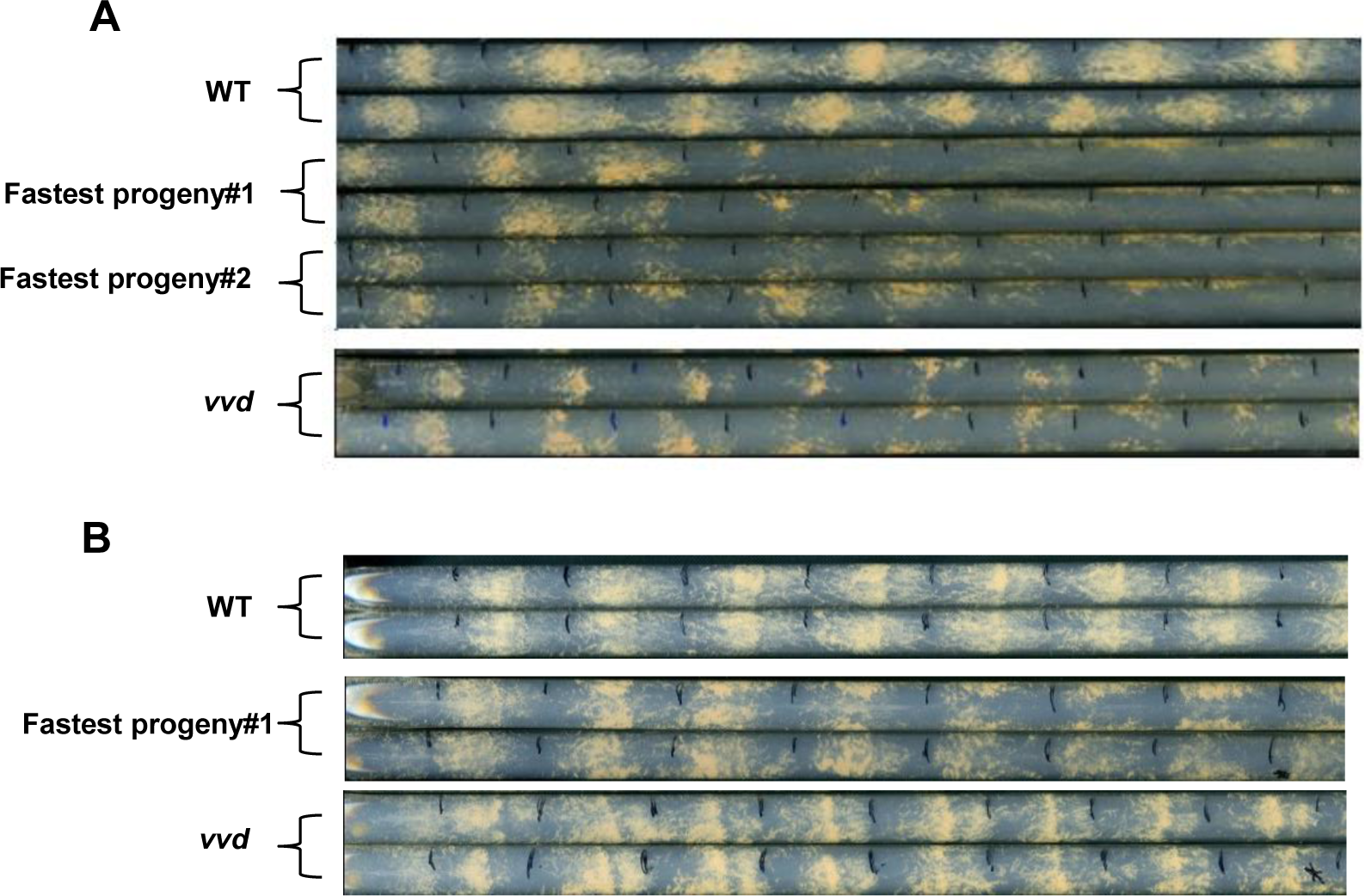

Supplement: S7 Fig — The vvd strain also showed dampening of rhythms when the light (blue) intensity was varied during the day phase from low (dawn) to high (mid-day/noon) and back to low (dusk) but with a maximum intensity of 10 μM m-2s-1. (B) Same experimental setup as in (A) but with the maximum intensity of 24 μM m-2s-1. (TIF) [file pgen.1005215.s007.tif]

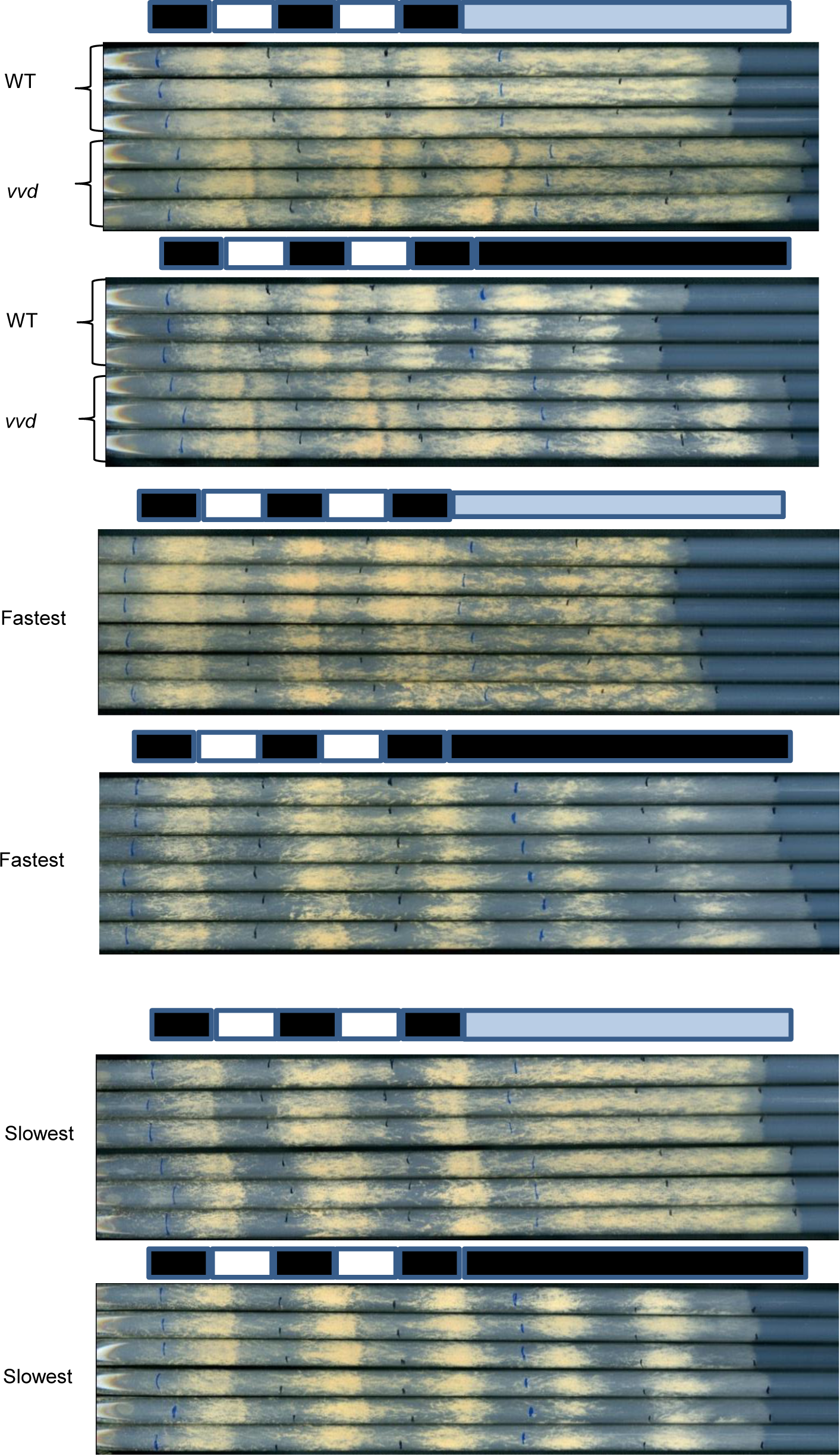

Supplement: S8 Fig — Race tubes used for densitometry in Fig 6. Strains were entrained for 2 days 12:12 LD cycles using bright blue light (~30 μM m-2s-1) before being released in either constant darkness or constant low light intensity blue light (~2μM m-2s-1). (TIF) [file pgen.1005215.s008.tif]

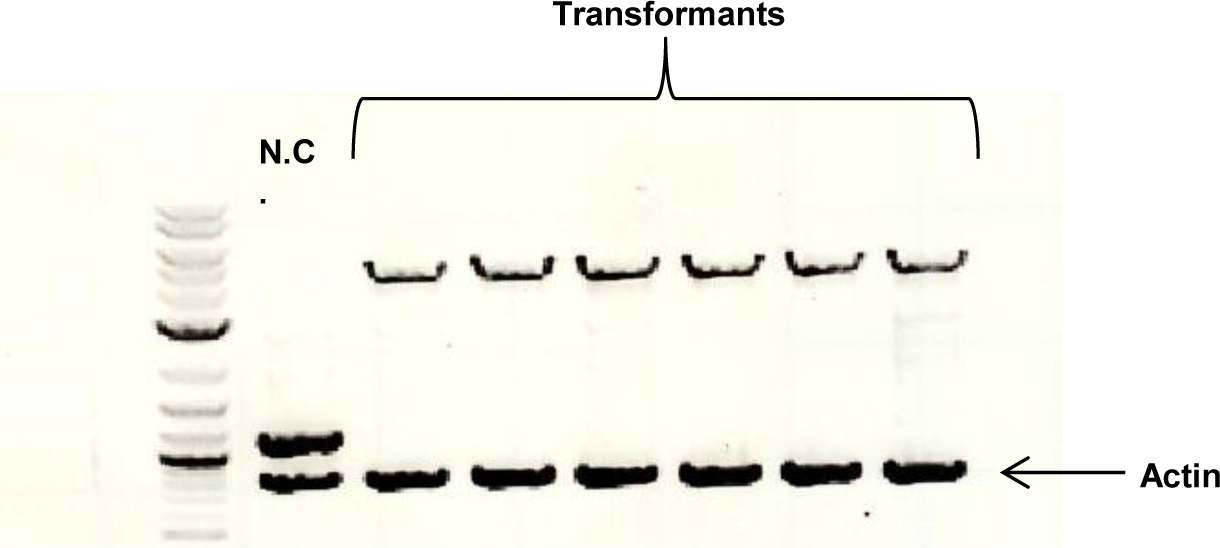

Supplement: S9 Fig — Actin was used as a control for amplification and primers to check integration were designed to sites flanking the site of integration. (TIF) [file pgen.1005215.s009.tif]
